# Highly porous copper-supported magnetic nanocatalysts: made of volcanic pumice textured by cellulose and applied for the reduction of nitrobenzene derivatives†

**DOI:** 10.1039/d1ra03538j

**Published:** 2021-07-21

**Authors:** Reza Taheri-Ledari, Mahdi Saeidirad, Fateme Sadat Qazi, Atefeh Fazeli, Ali Maleki, Ahmed Esmail Shalan

**Affiliations:** Catalysts and Organic Synthesis Research Laboratory, Department of Chemistry, Iran University of Science and Technology Tehran 16846-13114 Iran maleki@iust.ac.ir; BC Materials, Basque Center for Materials, Applications and Nanostructures, Martina Casiano, UPV/EHU Science Park Barrio Sarriena s/n Leioa 48940 Spain a.shalan133@gmail.com ahmed.shalan@bcmaterials.net; Central Metallurgical Research and Development Institute (CMRDI) P. O. Box 87, Helwan Cairo 11421 Egypt

## Abstract

Herein, a novel designed heterogeneous catalytic system constructed of volcanic pumice magnetic particles (VPMPs), cellulose (CLS) as a natural polymeric matrix, and copper nanoparticles (Cu NPs) is presented. Also, to enhance the inherent magnetic property of VPMP, iron oxide (Fe_3_O_4_) nanoparticles have been prepared and incorporated in the structure *via* an *in situ* process. As its first and foremost excellent property, the designed composite is in great accordance with green chemistry principles because it contains natural ingredients. Another brilliant point in the architecture of the designed composite is the noticeable porosity of VPMP as the core of the composite structure (surface area: 84.473 m^2^ g^−1^). This great porosity leads to the use of a small amount (0.05 g) of the particles for catalytic purposes. However, the main characterization methods, such as Fourier-transform infrared and energy-dispersive X-ray spectroscopy, thermogravimetric analysis, and electron microscopy, revealed that the spherical metallic particles (Fe and Cu oxides) were successfully distributed onto the surface of the VPMP and CLS matrices. Further, vibrating-sample magnetometer analysis confirmed the enhancement of the magnetic property (1.5 emu g^−1^) of the composite through the addition of Fe_3_O_4_ nanoparticles. Further, the prepared (Fe_3_O_4_@VPMP/CLS–Cu) nanocomposite has been applied to facilitate the reduction reaction of hazardous nitrobenzene derivatives (NBDs) to their aniline analogs, with 98% conversion efficiency in eight minutes under mild conditions. Moreover, the good reusability of the catalytic system has been verified after recycling it ten times without any significant decrease in the performance.

## Abstract 2

Herein, a novel designed heterogeneous catalytic system constructed of volcanic pumice magnetic particles (VPMPs), cellulose (CLS) as a natural polymeric matrix, and copper nanoparticles (Cu NPs) is presented.

## Graphical abstract

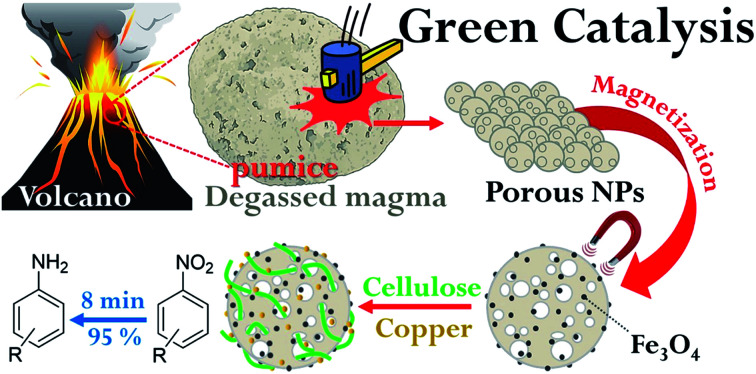

## 1. Introduction

As time goes on, microscale and nanoscale heterogeneous catalytic systems are gaining increasing attention for a variety of reasons, of which one of the most important is that they are able to create a high surface area for chemical reactions between the involved reactants and the catalytic substrate.^1–3^ In this regard, heterogeneous catalytic systems based on iron oxide nanoparticles have become more important due to the possibility of their easy separation by only holding an external magnet at the bottom of the reaction flask and decanting the content.^4–11^ Heterogeneous catalytic composites are modified by various components, such as organic structures, polymers, inorganic particles, biological structures, copper nanoparticles, and palladium nanoparticles, so that the majority of the catalytic sites are available.^12–20^ In recent years, more attention has been paid to green chemistry due to environmental concerns.^20,21^ That is why researchers are constantly trying to design novel natural heterogeneous catalytic systems with a high degree of biocompatibility. Undoubtedly, volcanic pumice benefits from a very porous structure that can lead to a high surface area, and it possesses properties such as high magnetic property, biocompatibility, and great surface functionalization capability; thus, it can inherently act as an eco-friendly substrate to create a heterogeneous catalytic composite.^21–25^

The use of cellulose (CLS) in this work, because it is natural, has been a strong indication of how important green chemistry was to us in this work.^26,27^ There are many reasons why cellulose, as a substantial polymeric substrate with a biocompatible origin, is used in this work; one of the most important of these is that cellulose, due to its large number of hydroxyl (–OH) functional groups, can form physicochemical hydrogen bonds with the –OH groups of pumice, which can lead to integration in the composite. On the other hand, the rest of the hydroxyl functional groups of cellulose, which are free of hydrogen bonds with the hydroxyl functional groups of pumice, are suitable for chelation by cationic metals such as copper and palladium and can form heterogeneous catalytic composites from natural components; this is one of the most important properties of this composite.^28^ The synthesized composite is notable even in terms of mechanical properties because it is a stable hybrid structure.^29,30^ As mentioned earlier, the synthetic heterogeneous catalytic composite in this work is suitable for scaling up and industrial applications because of its high biocompatibility and because it is made from components found in abundance in nature. In this work, after pumice, as an inorganic base with –OH groups functions as a suitable host for cellulose, as an organic base, Cu^2+^ ions are added to the system to act as catalytic sites to reduce the nitro functional groups of different compounds to amines after their conversion to Cu.^31,32^

Herein, an attempt was made to introduce a novel and convenient method for the synthesis of amine derivatives from the corresponding nitro compounds using Fe_3_O_4_@VPMP/CLS–Cu catalyst, which was synthesized with natural VPMP, CLS, and also Cu NPs. Then, the magnetic behavior and other critical characteristic properties of the prepared catalytic system, such as its average size, porosity, present chemical state, metallic elements, and thermal stability, were carefully investigated using different analytical methods. Then, the ability of this catalyst to reduce nitrobenzene derivatives (NBDs) was carefully examined. In summary, it was found that using the Fe_3_O_4_@VPMP/CLS–Cu catalytic system at 70 °C led to a 98% reaction yield in just 8 minutes. Also, the catalytic system under study can be reused for 10 successive runs due to its magnetic property, which leads to easy magnetic separation of this catalyst from the reaction medium. Also, in this study, water solvent was used as an eco-friendly solvent to evaluate the performance of the synthesized catalytic composite; this easily confirms the importance of green chemistry in this study.

## 2. Results and discussion

### 2.1. Preparation of the Fe_3_O_4_@VPMP/CLS–Cu nanocatalyst

**Scheme 1:**
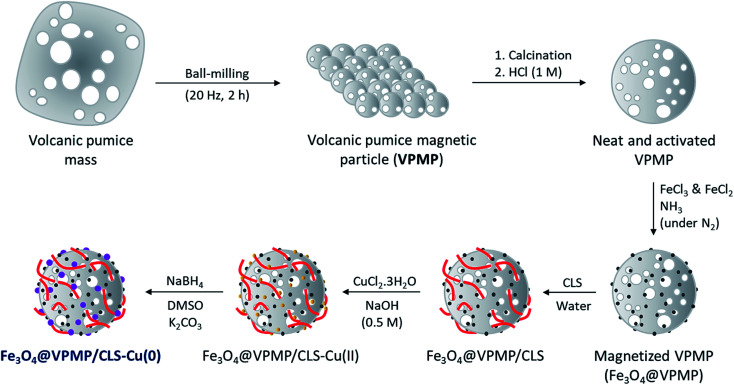
Preparation route of the Fe_3_O_4_@VPMP/CLS–Cu(0) nanocomposite.

As shown in Scheme 1, several steps are taken to prepare the Fe_3_O_4_@VPMP/CLS–Cu composite. Initially, the pumice is ground by a ball-mill to form uniform particles.^33^ After that, because the pumice is very porous and there may be useless fillers in its pores, it should be calcinated at a high temperature in a furnace.^34^ Then, if electron microscopy (EM) confirms that the calcinated pumice has uniformity and the useless fillers have been successfully removed from its pores, the magnetic property of the pumice is increased by Fe_3_O_4_ in order to separate it from the solvent on a laboratory scale, although the pumice itself has an inherent magnetic property. Next, it is it is dispersed and mixed in a concentrated solution of CLS under mild conditions. At the end of this step, the formed composite is magnetically separated and washed to remove the unbound CLS. Herein, a composite called Fe_3_O_4_@VPMP/CLS was prepared that contains a large number of hydroxyl functional groups in its VPMP and CLS structures and can be used several times. In another flask, an aqueous solution of CuCl_2_·H_2_O was prepared, which was added to a solution of dispersed Fe_3_O_4_@VPMP/CLS in which the hydroxyl functional groups were activated by an alkaline aqueous solution. After completion of the reaction, the resulting composite, Fe_3_O_4_@VPMP/CLS–Cu(ii), which was now brown and no longer black like magnetic pumice, was magnetically separated and washed to remove excess salts and ions trapped inside the pores and silicate network of the pumice. Ultimately, Cu(ii) in the composite of Fe_3_O_4_@VPMP/CLS–Cu(ii) was reduced to Cu(0) by sodium borohydride under alkaline conditions.^35^ Herein, we intend to monitor the catalytic ability of the Fe_3_O_4_@VPMP/CLS–Cu nanocatalyst in the reduction reactions of NBDs, and the results obtained from the mentioned experiments are reported in Table 1, in the optimization section.

**Table 1:** Optimization of the reduction reaction of nitrobenzene under various catalytic conditions

Entry	Cat.	Cat. (mg)	Cat. (mmol%)	NaBH_4_ (mmol)	Temp. (°C)	Time (min)	Yield (%)
1	—	—	—	—	80	180	N.R.
2	—	—	—	2	80	180	Trace
3	Fe_3_O_4_@VPMP/CLS	30	12	2	80	90	Trace
4	Fe_3_O_4_@VPMP/CLS–Cu	10	4	2	70	8	85
5	Fe_3_O_4_@VPMP/CLS–Cu	30	12	2	70	8	90
6	Fe_3_O_4_@VPMP/CLS–Cu	50	20	2	70	8	98^a^
7	Fe_3_O_4_@VPMP/CLS–Cu	60	24	2	70	8	98
8	Fe_3_O_4_@VPMP/CLS–Cu	50	20	3	70	8	94
9	Fe_3_O_4_@VPMP/CLS–Cu	50	20	1	70	8	90
10	CLS–Cu	50	20	2	70	8	92
11	Fe_3_O_4_@VPMP/CLS–Cu	50	20	2	R.t.	8	81
12	Fe_3_O_4_@VPMP/CLS–Cu	50	20	2	R.t.	30	97

a Optimum condition using the Fe_3_O_4_@VPMP/CLS–Cu nanocomposite (50.0 mg), nitrobenzene (1.0 mmol), NaBH_4_ (2.0 mol), and at 70 °C. Calculations related to mmol% of catalyst has been given in the ESI file. R.t. stands for room temperature.

### 2.2. Characterization of Fe_3_O_4_@VPMP/CLS–Cu nanocatalyst

#### 2.2.1. FTIR spectroscopy

**Fig. 1:**
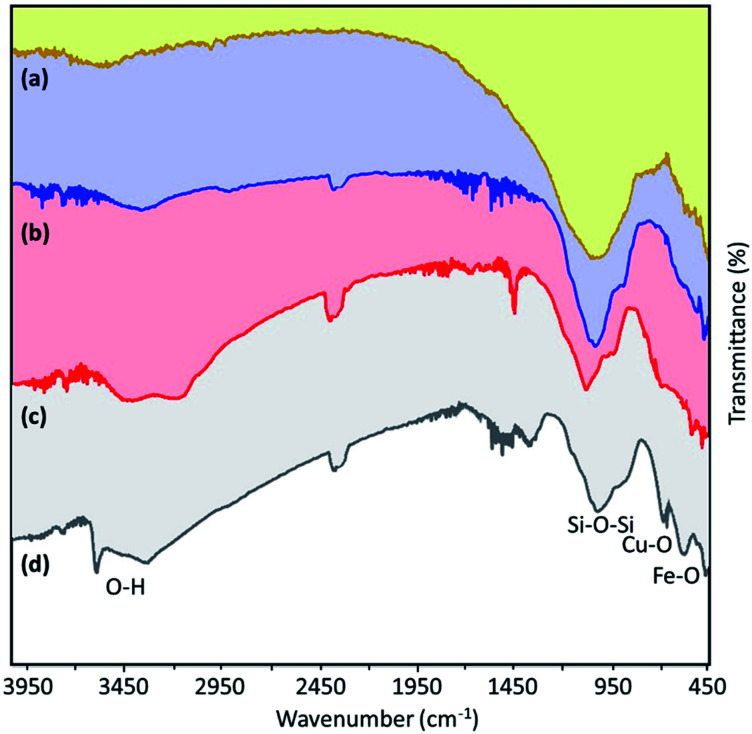
The FTIR spectra of (a) neat VPMP, (b) Fe_3_O_4_@VPMP nanoparticles, (c) Fe_3_O_4_@VPMP/CLS nanocomposite, and (d) Fe_3_O_4_@VPMP/CLS–Cu nanocomposite.

To examine the functional groups of the produced materials, FTIR spectra were obtained for the neat VPMP, Fe_3_O_4_@VPMP, Fe_3_O_4_@VPMP/CLS and Fe_3_O_4_@VPMP/CLS–Cu, as shown in Fig. 1. The results indicated that the existence of the peaks in all the materials spectra appearing at *ca.* 575–590 cm^−1^, 1000 cm^−1^ and 3450 cm^−1^ are related to the stretching vibrations of the metal oxide bonds (Fe–O), bending vibrations of the Si–O–Si bands of VPMP and stretching vibrations of the O–H bonds, respectively.^36^ Furthermore, the presence of Si–OH and SiO–H bonds was confirmed by the peaks that appeared at 950 cm^−1^ and 870 cm^−1^, respectively; these peaks are present in the spectra of all three samples.^37^ Finally, the peak that appeared at *ca.* 635 cm^−1^ well confirms the formation of Cu–O bonds in the structure of the Fe_3_O_4_@VPMP/CLS–Cu nanocomposite.^38^ The peak at 2300 cm^−1^ can also be related to CO_2_ in the atmosphere, and it is also visible in the spectra of all three samples.^39^

#### 2.2.2. EDX analysis

**Fig. 2:**
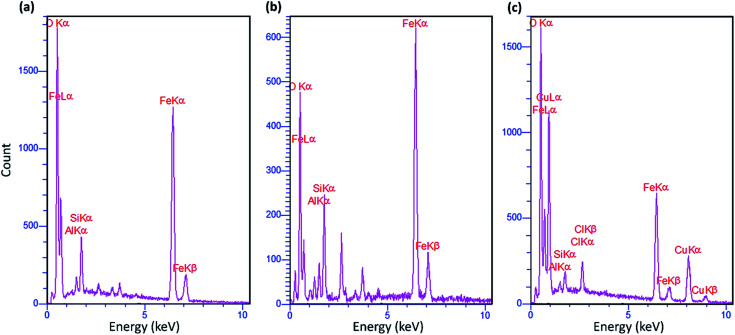
EDX spectra of (a) Fe_3_O_4_@VPMP, (b) Fe_3_O_4_@VPMP/CLS, and (c) Fe_3_O_4_@VPMP/CLS–Cu.

Energy-dispersive X-ray (EDX) spectroscopy should be used to further confirm the presence of elements that are predicted to be present at various stages of composite preparation. As shown in Fig. 2, all the composition elements for the three materials in the current study, Fe_3_O_4_@VPMP, Fe_3_O_4_@VPMP/CLS and Fe_3_O_4_@VPMP/CLS–Cu, were detected and affirmed through the EDX peaks with different intensities. Furthermore, the peak intensity of carbon element in the EDS of the fabricated Fe_3_O_4_@VPMP/CLS–Cu (Fig. 2a) increased significantly compared to that of the neat VPMP (Fig. 2b) and VPMP@CLS (Fig. 2c) samples, which is due to the good composition of CLS with VPMP in the presence of Cu nanoparticles in the magnetic nanocomposite structure. In addition, it has been clearly demonstrated that 10.1 wt% of the total weight of the fabricated Fe_3_O_4_@VPMP/CLS–Cu belongs to the Cu NPs.

#### 2.2.3. Electron microscopy

**Fig. 3:**
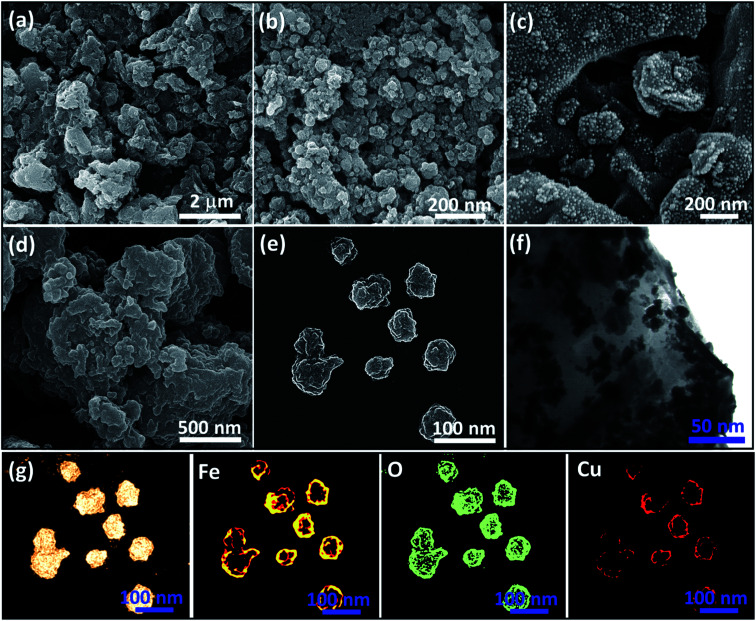
The SEM images of (a) ground pumice mass, (b) powdered VPMP, (c) Fe_3_O_4_@VPMP nanoparticles, (d) the integrated Fe_3_O_4_@VPMP/CLS composite, and (e) the dispersed Fe_3_O_4_@VPMP/CLS–Cu nanocomposite; (f) the TEM image of the Fe_3_O_4_@VPMP/CLS–Cu nanocomposite; and (g-series) SEM energy-mapping images of the Fe_3_O_4_@VPMP/CLS–Cu nanocomposite, related to image (e).

In order to investigate the size, morphology, and dispersion state of the prepared Fe_3_O_4_@VPMP/CLS–Cu nanocomposite, scanning-electron microscopy (SEM) and transmission-electron microscopy (TEM) were used. As can be observed in Fig. 3a, the ground VPMP was obtained from the pumice mass, but there was no uniformity in size or shape. Also, the size of the ground particles is in the range of 500–1100 nm, which is not appropriate. Fig. 3b illustrates the powdered VPMP (obtained by ball-milling), which has high uniformity of its size and morphology. As can be seen, good dispersion of the spherical-shaped VPMP particles with the average sizes of *ca.* 64 nm have been obtained. Also, with a more accurate look at the image (b), it is recognized that the rough structure of the VPMP nanoparticles is quite porous. Fig. 3c exhibits the composition state of the iron oxide (Fe_3_O_4_) nanoparticles with the VPMP nanoparticles. It can be clearly observed that the *in situ* preparation and incorporation of Fe_3_O_4_ were successfully performed, as the Fe_3_O_4_ seeds with a mean size of *ca.* 27 nm are finely distributed on the VPMP surfaces. Moreover, the Fig. 3g-series demonstrates the SEM energy-mapping images of the Fe_3_O_4_@VPMP nanoparticles that are related to image (e). Fig. 3d shows that the Fe_3_O_4_@VPMP/CLS particles are slightly agglomerated due to the integration of the CLS polymeric matrix. In this stage, good coating of Fe_3_O_4_@VPMP by the CLS occurred, and the particle agglomeration is temporary because the Fe_3_O_4_@VPMP/CLS particles were further redispersed in an alkaline medium (NaOH, 0.5 M) and also washed several times. As can be observed in Fig. 3e, good dispersion of the Fe_3_O_4_@VPMP/CLS–Cu nanocomposite was obtained through the washing of the particles and functionalization with the copper ions. Actually, the excess CLS matrix was removed through re-dispersion *via* the ultrasonication and additional washing. As can be seen in image (e), the uniformity of the particles (in size and shape) was preserved during the copper-functionalization process. Furthermore, the mean size of the Fe_3_O_4_@VPMP/CLS–Cu particles was estimated to be *ca.* 81 nm, which is good for catalytic applications. Ultimately, Fig. 3f illustrates the TEM image of the Fe_3_O_4_@VPMP/CLS–Cu composite, in which the dark spots are attributed to the Fe_3_O_4_ and Cu nanoparticles on the surface of the VPMP/CLS substrate. The energy-mapping images of the Fe_3_O_4_@VPMP/CLS–Cu composite related to image (e) have been illustrated as the “g series”, demonstrating the presence of Cu element on the surfaces. Generally, there are two justifications for incorporation of the Cu nanoparticles into the VPMP/CLS matrix; first, there are strong metal–oxide (M–O) electronic interactions between the Cu nanoparticles and abundant hydroxyl groups in the structure of CLS.^40^ Second, the Cu nanoparticles are incorporated into the voids of the VPMP, which is a highly porous substrate.^41^

#### 2.2.4. VSM analysis

**Fig. 4:**
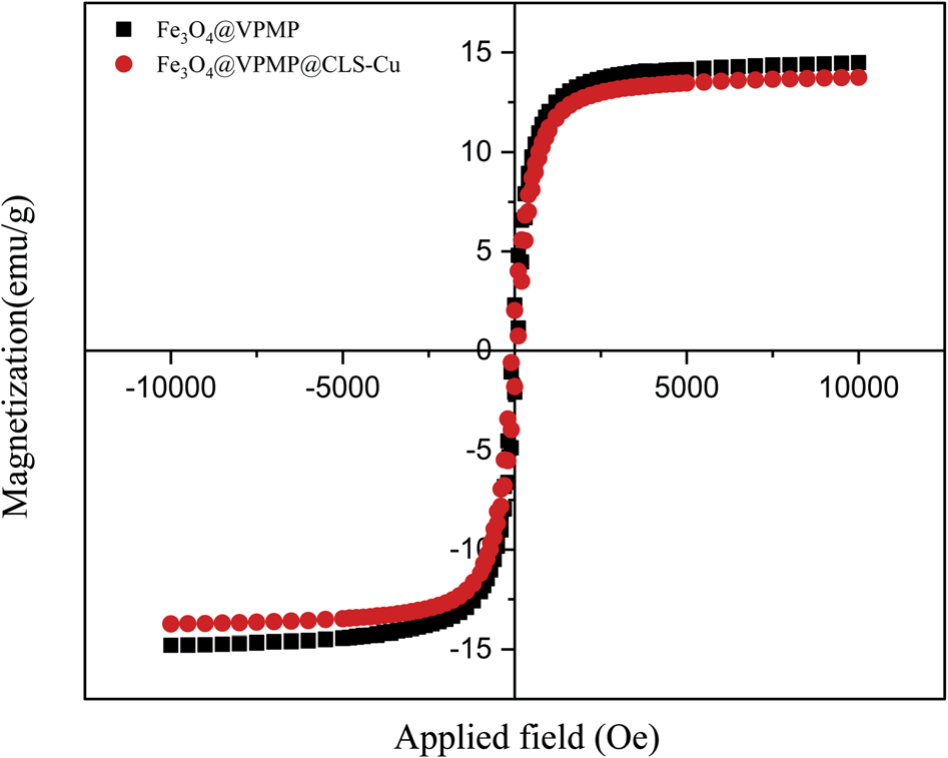
The VSM curves of Fe_3_O_4_@VPMP and Fe_3_O_4_@VPMP/CLS–Cu.

The vibrating-sample magnetometer (VSM) technique was applied to detect the intrinsic magnetic performance of the arranged Fe_3_O_4_@VPMP and Fe_3_O_4_@VPMP/CLS–Cu nanocomposite materials. The hysteresis curves of the Fe_3_O_4_@VPMP nanocatalyst exhibit about a *ca.* 10.0 emu g^−1^ decrease in magnetic saturation, which is related to the phenomenon in which not all of the nanocatalysts were found to present magnetic features (Fig. 4). As is perceived from the magnetic–hysteresis curves, the magnetic behavior of the Fe_3_O_4_@VPMP nanocomposite has not been considerably reduced after addition of the CLS–Cu particles to form Fe_3_O_4_@VPMP/CLS–Cu nanocomposite materials. Additionally, the Fe_3_O_4_@VPMP/CLS–Cu nanocomposite materials could be collected, through this excellent magnetic property, without difficulty *via* using a magnet, and they could be reused several times.

#### 2.2.5. TGA/DTA analysis

**Fig. 5:**
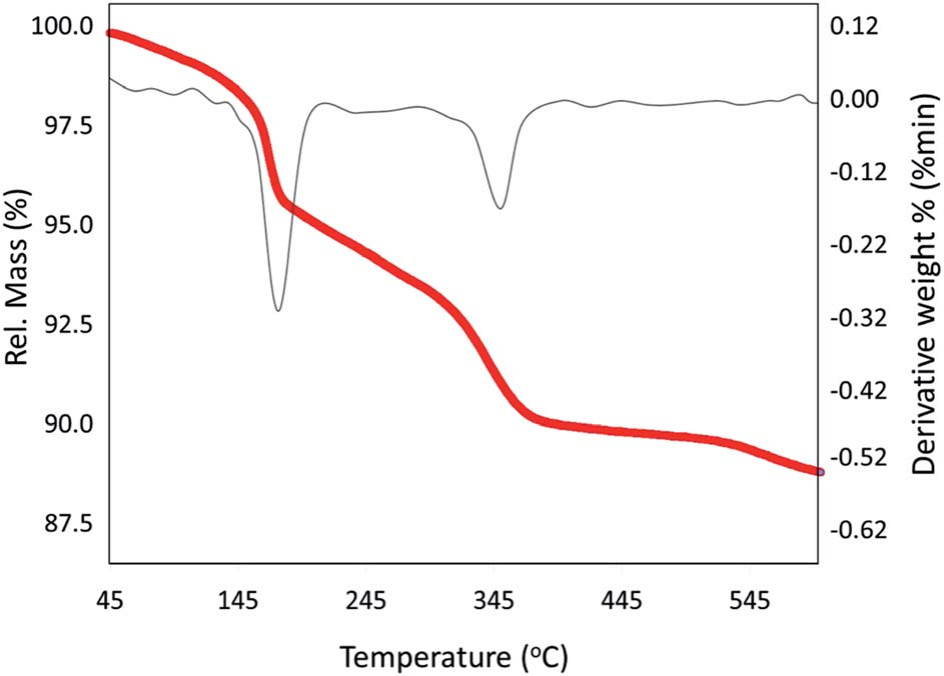
The TGA (red) and DTA (gray) curves of the fabricated Fe_3_O_4_@VPMP/CLS–Cu nanocomposite.

In this section, the thermal decomposition of the neat VPMP and the fabricated Fe_3_O_4_@VPMP/CLS–Cu system is investigated by thermogravimetric analysis (TGA) and differential thermal analysis (DTA). As shown in Fig. 5, in the temperate range of 150–370 °C, about 10% of the mass of Fe_3_O_4_@VPMP/CLS–Cu (black curve) was lost, while this reduction in mass for the neat VPMP from 300 °C to 500 °C is about 5%. This mass reduction in the TGA curve of Fe_3_O_4_@VPMP/CLS–Cu is related to the removal of –OH groups in the CLS structure. According to the literature, the –OH groups of polymers leave the structure during the dehydration process at this thermal range. Also, this change of mass due to the removal of the –OH groups of CLS in the DTA curve shows itself as an endotherm. Above 400 °C, both the Cu nanoparticles and VPMP NPs begin to decompose, where the reduction in mass of the prepared composite is very slow. This mass reduction in the DTA curve is shown as an endotherm. The silicate network has a high ability and capacity to trap various molecules, including water molecules, in its structure; therefore, it seems that the 5% reduction in the neat VPMP mass is probably related to the removal of water molecules trapped in the silicate structure of the VPMP structure.^42^ In addition, the first shoulder in the TGA curve of neat VPMP is about 1 wt%, and it is related to the separation of physically adsorbed moisture onto the surfaces. However, this shoulder is not in the TGA curve of Fe_3_O_4_@VPMP/CLS–Cu, which seems to be due to the good drying of the Fe_3_O_4_@VPMP/CLS–Cu sample under N_2_ before TGA. According to the DTA curve, the studied nanocomposite maintains its structure up to 600 °C, and no melting or crystallization action occurs up to the mentioned temperature in its structure (Fig. 5).

#### 2.2.6. XRD analysis

**Fig. 6:**
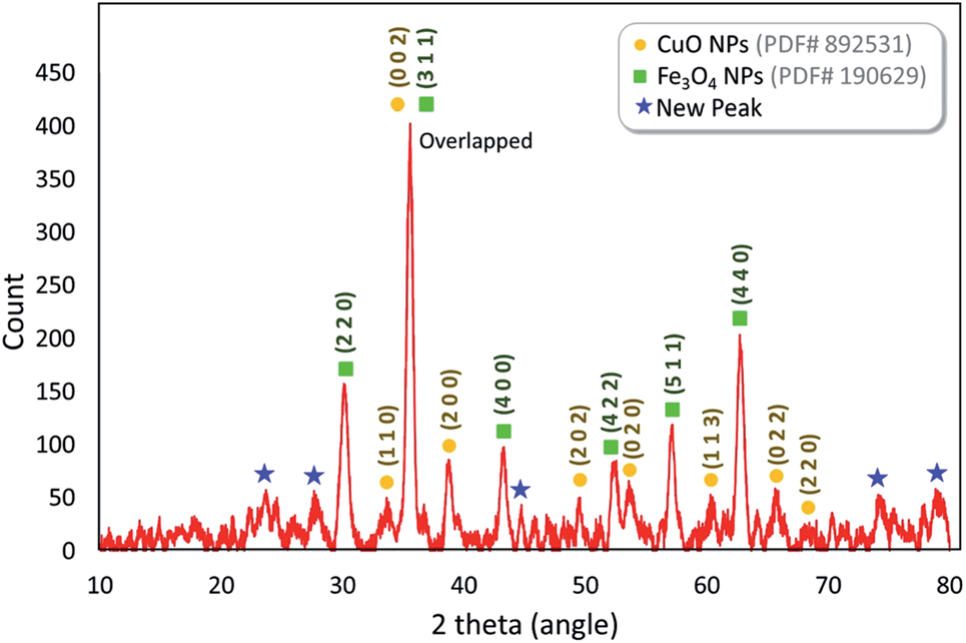
The XRD pattern of the fabricated Fe_3_O_4_@VPMP/CLS–Cu nanocomposite in comparison with the reference patterns of CuO and Fe_3_O_4_ NPs.

The X-ray diffraction pattern (XRD) for the Fe_3_O_4_@VPMP/CLS–Cu compound is shown in Fig. 6. In this regard, the pattern of this nanocomposite was compared with the separate patterns of Fe_3_O_4_ and CuO based on the JCPDS database (PDF # 892531 and PDF # 190629). According to the results, the peaks found at 2*θ* = ∼30.7°, 36.2°, 43.8°, 52.2°, 57.7°, and 63.4°, which are respectively assigned to the green Miller indices (2 2 0), (3 1 1), (4 0 0), (4 2 2), (5 1 1), and (4 4 0), are related to the XRD patterns of Fe_3_O_4_ magnetic nanoparticles (MNPs). Also, the peaks that appeared at 2*θ* = ∼33.1°, 38.8°, 49.3°, 53.8°, 61.7°, 68.7°, and 69°, which were respectively assigned to the yellow Miller indices (1 1 0), (0 0 2), (2 0 0), (2 0 2), (0 2 0), (1 1 3), (0 2 2) and (2 2 0), in Fig. 6 are related to the diffraction pattern of the CuO structure in the composite of Fe_3_O_4_@VPMP/CLS–Cu.^43^ Also, the new peaks appearing at 2*θ* = ∼22.3°, 23.6°, 44.6°, 74.2°, and 79.6° are related to the amorphous structure of the VPMP structure.

#### 2.2.7. BET analysis

**Fig. 7:**
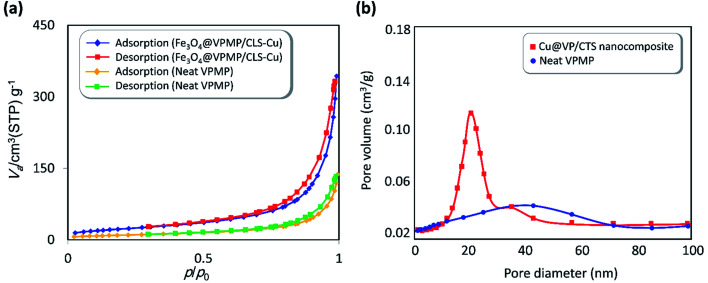
(a) The BET curves of the neat VPMP and the fabricated Fe_3_O_4_@VPMP/CLS–Cu nanocomposite and (b) the mean pore volumes of the neat VPMP and the fabricated Fe_3_O_4_@VPMP/CLS–Cu nanocomposite, *via* adsorption/desorption of N_2_ under ambient conditions.

Due to the highly porous structure of the pumice used in the synthesis of the Fe_3_O_4_@VPMP/CLS–Cu nanocomposite, adsorption/desorption of N_2_ gas Brunauer–Emmett–Teller (BET) surface area analysis was used to confirm the porosity of the synthesized composite, as shown in Fig. 7. According to the obtained results, the pumice structure and Fe_3_O_4_@VPMP/CLS–Cu nanocomposite indicate the type IV isotherm of mesoporous materials. According to the obtained curves, all the pores of Fe_3_O_4_@VPMP/CLS–Cu were filled by liquid N_2_ at *P*/*P*_0_=0.99; therefore, for our sample, according to the amount adsorbed at *P*/*P*_0_ = 0.99 (72.2 cm^3^ g^−1^ STP) and its conversion to liquid volume (gas per liquid volume ratio is 647 for N_2_ at 77 K), we can estimate that the total volume of the composite pores is 0.5164 cm^3^ g^−1^. Also, according to the results, the reduction of the average pore size from 40 nm for pumice powder to 24.452 nm in the Fe_3_O_4_@VPMP/CLS–Cu nanocomposite along with the total surface area of the nanocomposite, which is 84.473 m^2^ g^−1^, confirms that although a large number of pumice pores are filled with Fe_3_O_4_ and CLS–Cu magnetic nanoparticles, large internal pores are still available for the catalytic performance of the nanocomposite.

#### 2.2.8. XPS analysis

**Fig. 8:**
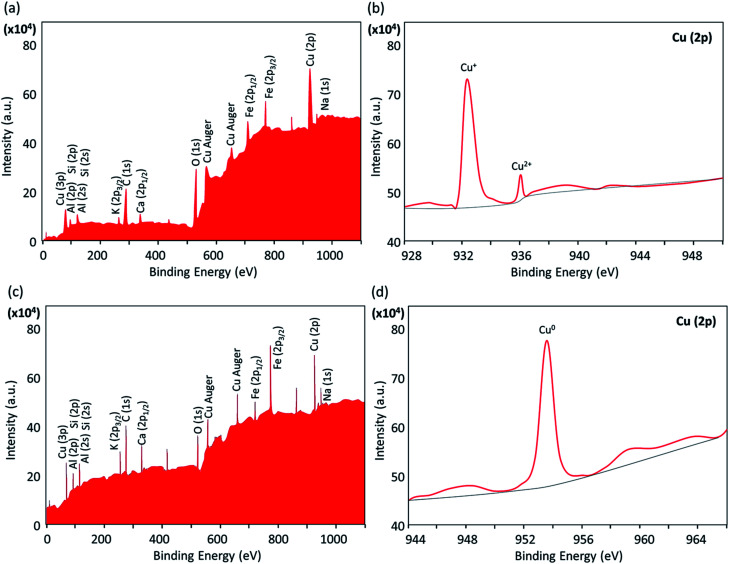
The XPS spectra of the prepared Fe_3_O_4_@VPMP/CLS–Cu nanocomposite: (a and b) before and (c and d) after reduction by NaBH_4_; (a and c) standard form and (b and d) expanded form of a specific area.

In order to evaluate the chemical state of the copper nanoparticles (Cu^0^, Cu^+^ or Cu^2+^) in the structure of the composite, X-ray photoelectron spectroscopy (XPS) was used. As demonstrated in Fig. 8a, the presence of Al/Si, Fe, and Cu elements in the structure of the Fe_3_O_4_@VPMP/CLS–Cu nanocomposite was confirmed; these elements originate from the VPMP, Fe_3_O_4_ and Cu nanoparticles, respectively. In the expanded spectrum of the copper element related to Cu 2p (Fig. 8b), there are two peaks at *ca.* 933 and 936 eV marked by Cu^+^ and Cu^2+^, respectively, confirming the formation of Cu_2_O and CuO nanoparticles in the structure of the catalyst.^44^ Therefore, it is concluded that the stage of reduction with sodium borohydride is required before beginning the catalytic process for the nitrobenzene derivatives. Also, the chemical state of the copper particles after the reduction process by sodium borohydride in alkaline conditions was investigated by this method. As can be observed in Fig. 8c and d, the peak of the copper element (Cu 2p) appeared at *ca.* 953 eV, confirming the chemical state Cu^0^ after the reduction process.^45^

### 2.3. Application of Fe_3_O_4_@VPMP/CLS–Cu nanocatalyst in the reduction reactions of nitrobenzene derivatives (NBDs)

#### 2.3.1. Optimization of the catalytic system in the reduction of nitrobenzene

In order to determine the optimized conditions for the use of the Fe_3_O_4_@VPMP/CLS–Cu catalytic system, the catalytic amount of the nanocomposite, amount of NaBH_4_, medium, temperature and reaction time used in the reduction reaction of nitrobenzene (NB) must be carefully monitored. Details of this investigation are reported in the table below. As shown in the table, in order to prove the important role of pumice porosity in the catalytic activity of Fe_3_O_4_@VPMP/CLS–Cu, CLS–Cu alone was applied under the same conditions during the reduction reaction, which showed a reduction in the reaction yield. This confirms the role of the pumice porosity in the catalytic activity of Fe_3_O_4_@VPMP/CLS–Cu. Also, the role of copper as the main catalytic site was investigated, which, as predicted, showed that the non-use of copper in the catalytic system leads to a sharp decrease in the yield of the reaction. From the table, it has been revealed that the optimum conditions were provided by using 0.05 g of the Fe_3_O_4_@VPMP/CLS–Cu catalyst during 8 min of stirring at 70 °C.

#### 2.3.2. Catalyzed synthesis of the aniline derivatives

In order to study the catalytic efficiency of the prepared Fe_3_O_4_@VPMP/CLS–Cu composite, NBDs were utilized, and the results are shown in Table 2. Interestingly, by using the Fe_3_O_4_@VPMP/CLS–Cu catalytic system, in a short time, a high yield reaction was obtained from the reduction reactions of NBDs, which proves that the Fe_3_O_4_@VPMP/CLS–Cu catalytic system is a promising candidate for reduction of NBDs compared to other catalysts. In addition, the ^1^H-NMR and ^13^C-NMR spectra and spectral data of aniline as well as its derivatives are founded in the ESI file (Fig. S1–S20†).

**Table 2:** Gained yields after the reduction of NB derivatives to aniline analogues under the optimized conditions

Entry	NB structure	Product	Time (min)	Yield (%)	Mp (°C)	
					Found	Reported
1	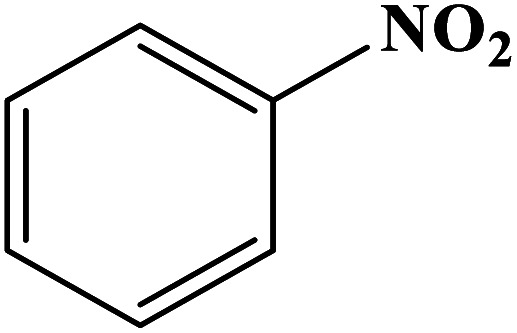	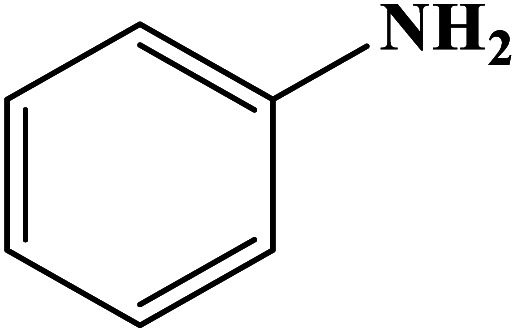	8	98	Liquid sample	(Ref. 46)
2	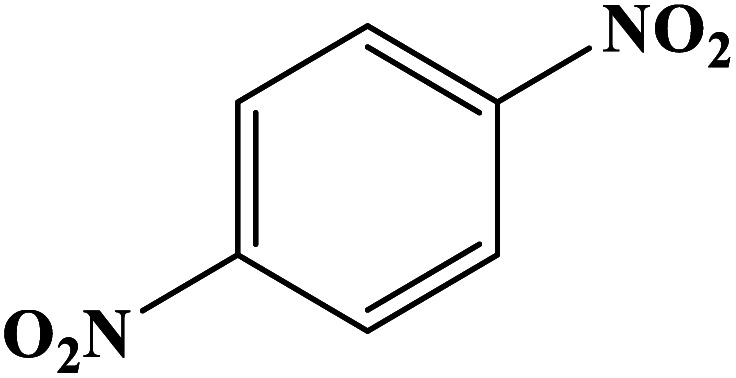	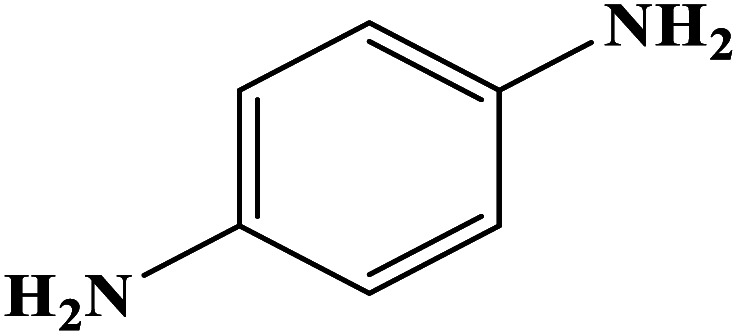	13	97	145–146	144–146 (ref. 47)
3	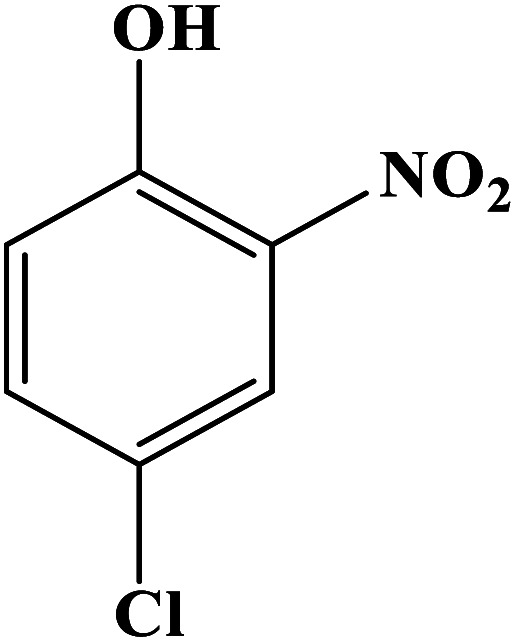	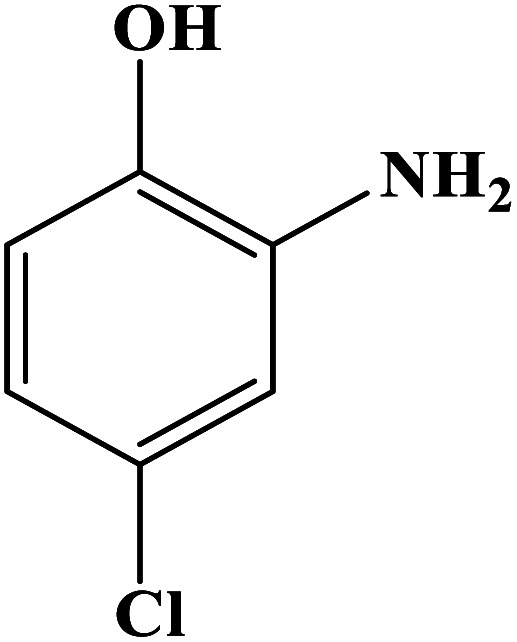	7	96	135–138	136–138 (ref. 48)
4	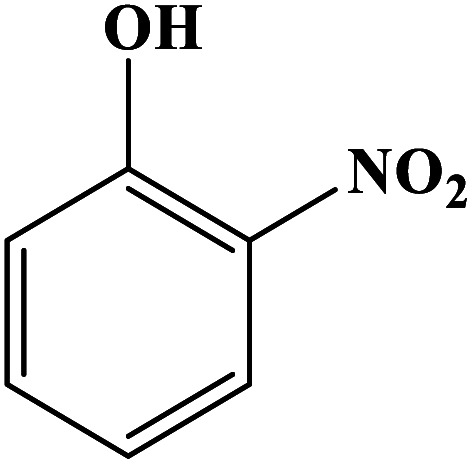	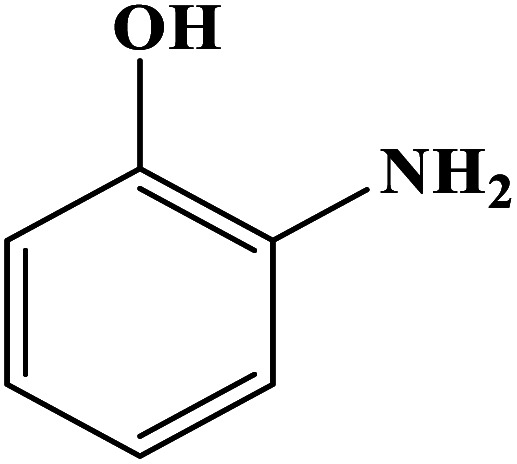	3	98	172–175	171–173 (ref. 49)
5	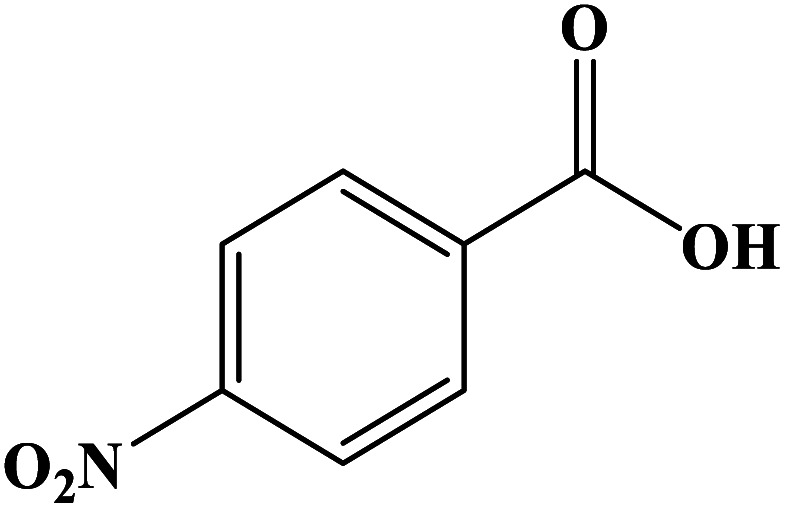	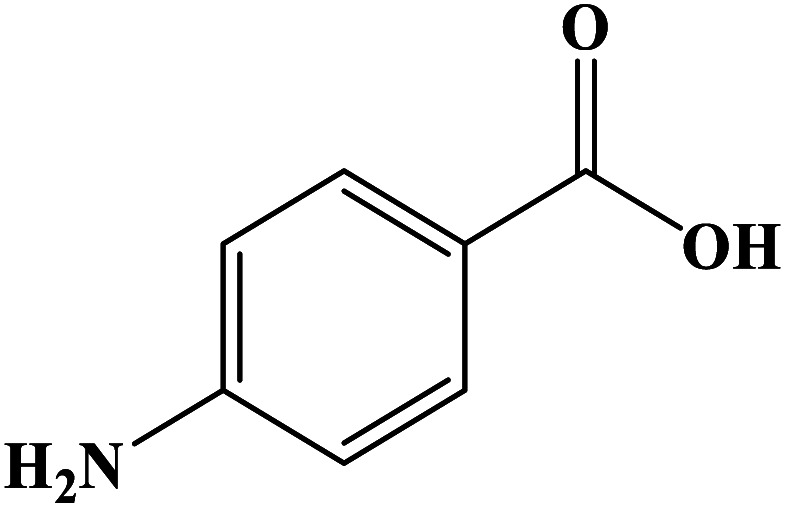	4	96	187–189	186–188 (ref. 46)
6	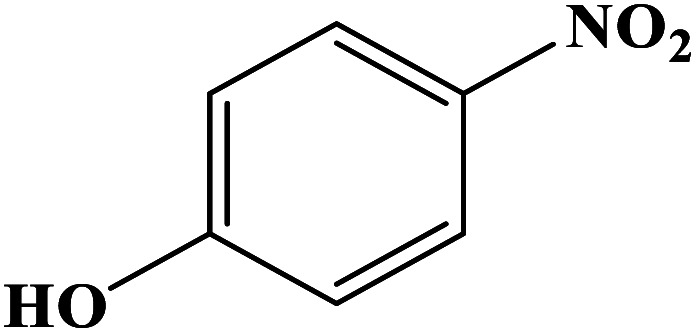	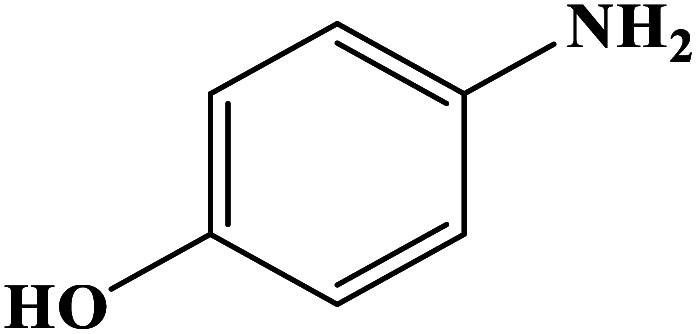	8	95	184–186	185–187 (ref. 49)
7	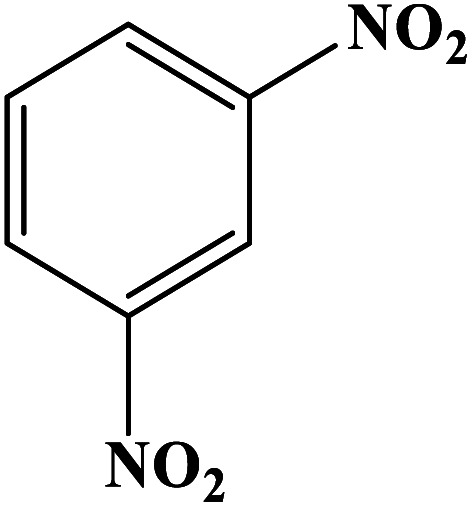	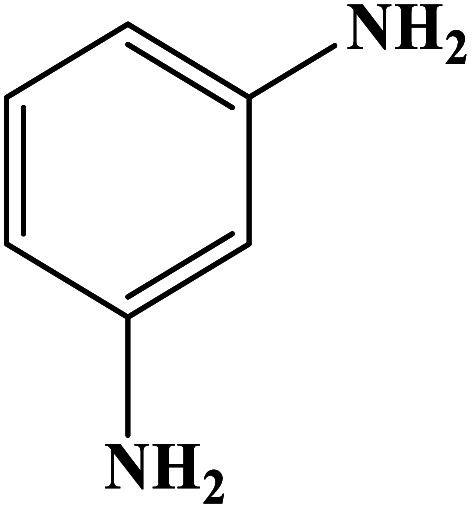	13	89	65–66	65–66 (ref. 50)
8	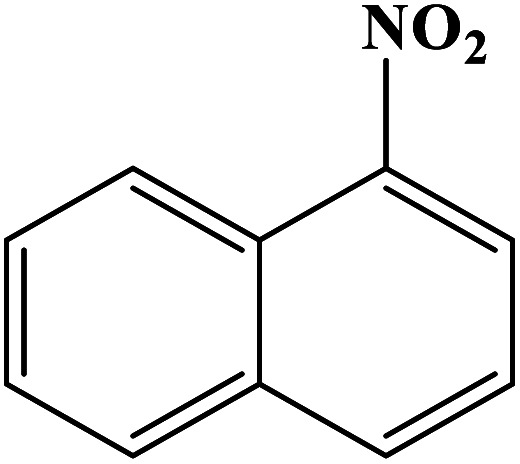	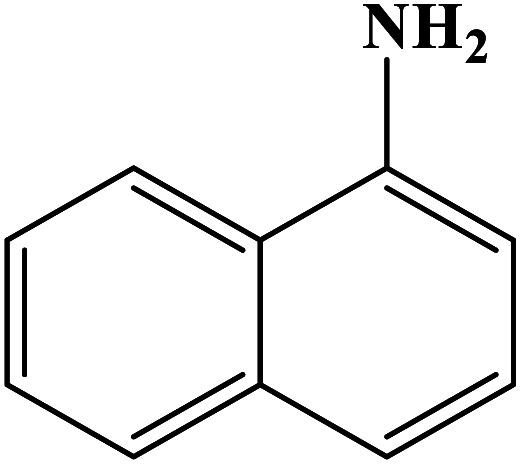	12	86	51–53	52–53 (ref. 51)
9	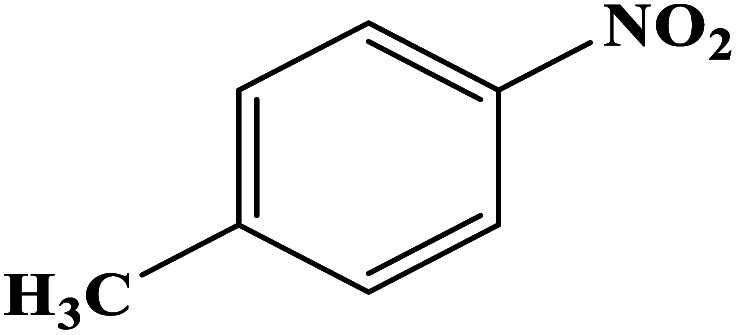	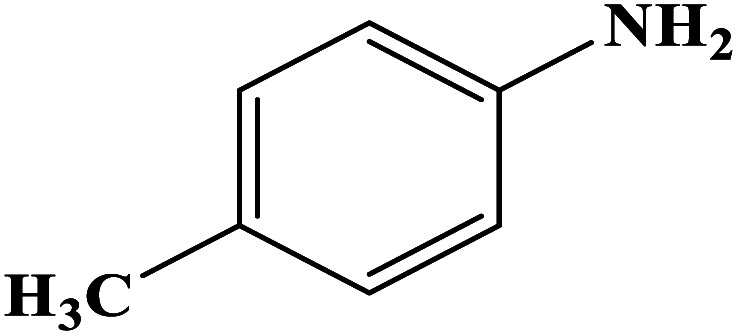	7	97	42–44	42–43 (ref. 52)
10	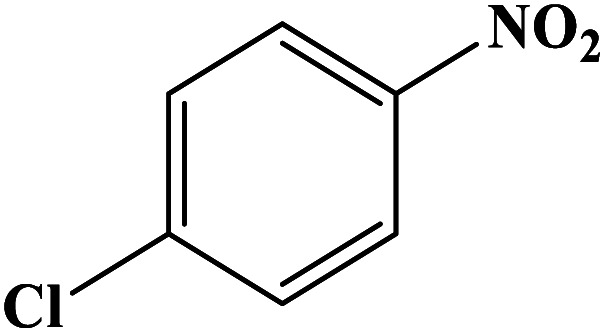	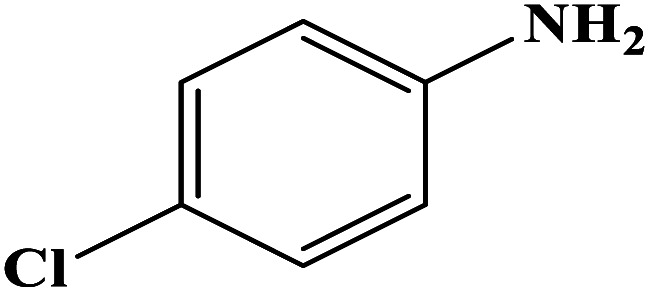	9	97	70–71	70–72 (ref. 53)

#### 2.3.3. Suggested mechanism

**Scheme 2:**
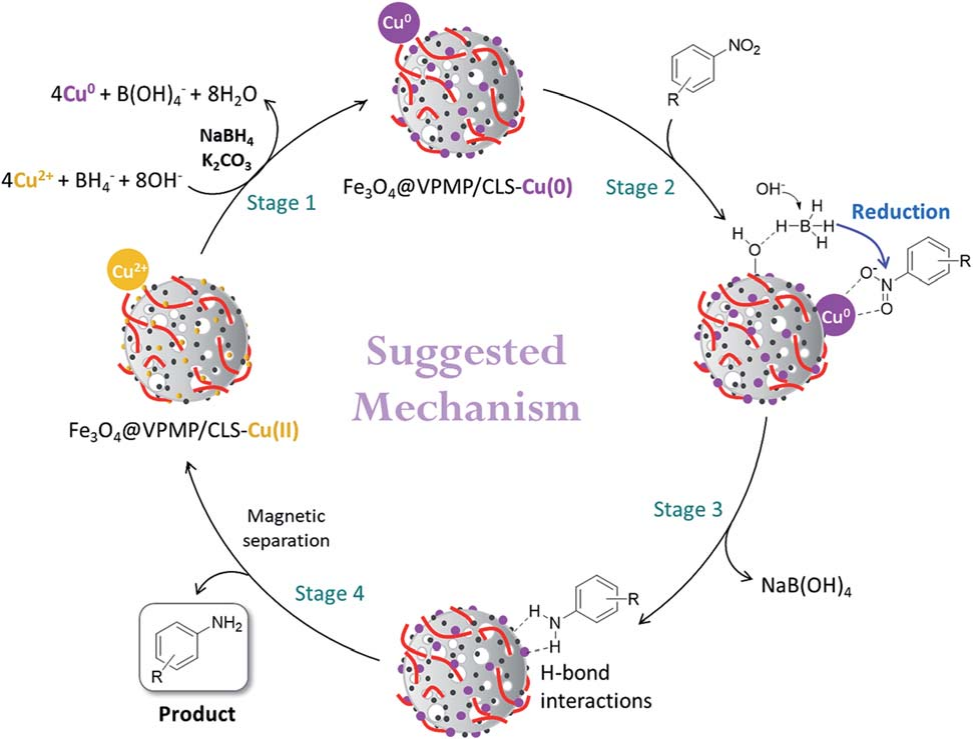
A plausible multi-stage mechanism for the reduction of the NBDs catalyzed by the fabricated Fe_3_O_4_@VPMP/CLS–Cu catalytic system.

As shown in Scheme 2, a plausible mechanism can be suggested for the reduction process of the NBDs under catalytic conditions provided by the Fe_3_O_4_@VPMP/CLS–Cu nanocomposite. At the first stage, the Cu^2+^ ions as the main catalytic sites are reduced to Cu^0^ by sodium borohydride (NaBH_4_) under the alkaline conditions provided by potassium carbonate (K_2_CO_3_).^54^ In stage 2, both the NB and borohydride (BH_4_^−^) structures are attracted onto the catalyst surface through electronic and hydrogen bond interactions.^55^ In third stage, hydridation of the NB structure is performed by the borohydride in the vicinity of the NB on the surface of the Fe_3_O_4_@VPMP/CLS–Cu particle. In the alkaline conditions, sodium metaborate (NaB(OH)_4_) is produced after completion of the reduction reaction of the NB. Finally, in stage 4, the produced aniline structure leaves the surface of the catalyst, and the particles are magnetically separated from the mixture, washed, and reused several times.

#### 2.3.4. Recyclability effect of the nanocomposite catalytic system

**Fig. 9:**
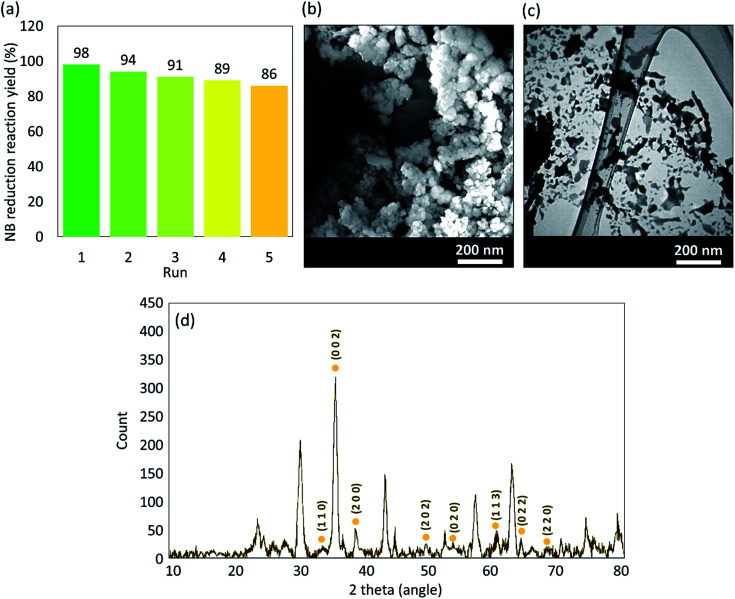
(a) Recyclability diagram of the Fe_3_O_4_@VPMP/CLS–Cu catalytic system in the reduction reaction of NB to aniline; (b) SEM image, (c) TEM image, and (d) XRD pattern of the recovered Fe_3_O_4_@VPMP/CLS–Cu particles after recycling five times.

In order to evaluate the reusability of the prepared Fe_3_O_4_@VPMP/CLS–Cu catalytic system, a reasonable investigation was performed on the model reduction reaction of nitrobenzene (NB). Firstly, the half-life of the reaction was estimated in five successive runs of the reaction. The details of the experiment and the obtained results are reported in Table S1 and Fig. S21, ESI file.† As reported in Table S2 (in the ESI file†), the time of the half-life of the reduction reaction of the NB changed to higher values after each cycle of separation and re-preparation of the catalyst particles. Based on the obtained results, it can be concluded that the catalytic efficiency of the Fe_3_O_4_@VPMP/CLS–Cu system is gradually lost during the recycling and reusing processes. To investigate the possible reasons, probable leaching of the copper particles from the Fe_3_O_4_@VPMP/CLS–Cu system as the main catalytic site was checked by inductively coupled plasma mass spectrometry (ICP-MS) analysis of the supernatant after the separation of the magnetic particles after running each recycling process. As can be seen in Table S3 (in the ESI file†), a small amount of leaching of the copper particles occurred during the separation, rinsing, drying and reusing of the Fe_3_O_4_@VPMP/CLS–Cu particles in five successive runs. Most likely, the copper leaching occurred during the ultrasonication process that is performed for dispersion of the particles before each run. The resulting reaction yields during five-time reuse of the Fe_3_O_4_@VPMP/CLS–Cu particles are given in the bar chart in Fig. 9a. As can be seen, it can be concluded that the Fe_3_O_4_@VPMP/CLS–Cu particles can be recovered and reused at least five successive times after magnetic separation, rinsing, and drying of the particles. Obviously, this has economic benefits, particularly in large scale applications. In order to investigate any possible change in the structure of the Fe_3_O_4_@VPMP/CLS–Cu nanocatalyst after five-time recycling, SEM and TEM imaging of the recovered particles were carried out. As illustrated in Fig. 9b and c, the uniformity and integrity of the VPMP, Fe_3_O_4_ and Cu nanoparticles in the CLS matrix have been suitably maintained, which confirms the good physicomechanical stability of the Fe_3_O_4_@VPMP/CLS–Cu composite. Also, the crystalline structure of the recovered Fe_3_O_4_@VPMP/CLS–Cu composite was studied by XRD spectroscopy. As can be observed in Fig. 9d, the same pattern with the fresh catalyst (Fig. 6) was obtained after recycling five times; only the intensity of the peaks related to the CuO nanoparticles (marked with Miller indices) decreased slightly due to the copper leaching. The content of Cu in the recovered structure was evaluated by inductively coupled plasma optical emission spectrometry (ICP-OES) analysis, and the obtained results, which have been given in Table S4 (in the ESI†), confirm the Cu-leaching from the catalytic system during the recycling. Besides, the graph of the optimization stages of reduction reaction of nitrobenzene under various catalytic conditions (related to Table 1) is illustrated in Fig. S22, ESI file.†

#### 2.3.5. Comparison of the prepared Fe_3_O_4_@VPMP/CLS–Cu with other catalytic systems

In this section, we attempt to make a quick comparison between our presented nanocatalyst and a number of other previously reported systems that, like the Fe_3_O_4_@VPMP/CLS–Cu catalyst, have the ability to convert NBDs to their aniline analogues. Fe_3_O_4_@VPMP/CLS–Cu has advantages over the other catalysts discussed in the table below: (1) in general, heterogeneous catalytic systems are preferred to homogeneous catalytic systems due to their convenient separation process. The VPMP, which is used in our presented nanocatalyst, has a magnetic property itself due to the presence of iron in its structure; however, to increase this property, Fe_3_O_4_ was added to the synthetic composite, which leads to easier separation of this catalytic composite from the reaction medium. Significant conversion (98%) of different NB derivatives occurred in a short time (often less than 10 minutes), while in most cases (entries 1, 3, 4 and 5), the reaction time exceeded 90 minutes. In the design and preparation phase, it is very important to use materials that, in addition to benefiting from high degrees of biocompatibility, are inexpensive; therefore, the Fe_3_O_4_@VPMP/CLS–Cu composite, due to the use of abundant natural materials (such as VP rock), which has many economic advantages, can be considered superior to the systems reported in the table below (entry 2), although the results obtained by the reported catalytic systems are excellent. In addition to the mentioned advantages, other advantages of this catalytic system are easy preparation of the composite in question and use of lightweight substrates with a porous structure, which leads to good dispersion of the catalyst particles in the reaction medium and an overall increase in performance (Table 3).

**Table 3:** Catalytic systems previously reported for reduction of NB derivatives to aniline analogues

Entry	Catalyst	Conditions	Cat. (mg)	Time (min)	Conversion (%)	Ref.
1	Ag–MPTA-1^a^	80 °C/NaOH/N_2_	250	600	97	56
2	Ag–SBA-15^b^	R.t./NaBH_4_	10	6	95	57
3	Nano Co^c^	R.t./N_2_H_4_·H_2_O	5.6	30	50	58
4	PhNO_2_,Pd NPs/RGO^d^	50 °C/NaBH_4_	6.0	90	98	59
5	Cu NPs^e^	50 °C	6.0	120	95	60
6	Fe_3_O_4_@VPMP/CLS–Cu^f^	70 °C	5.0	8	98	—

a Poly-triallylamine (MPTA-1).

b SBA-15 is a mesoporous silica.

c Cobalt nanoparticles.

d Reduced graphene oxide.

e Copper nanoparticles.

f The present work.

## 3. Experimental

### 3.1. Chemicals and instruments

All the chemicals, reagents, and equipment used in this study are listed in Table 4.

**Table 4:** Chemicals and equipment used in this study

Materials and equipment	Purity and brand
Pumice stone	Granulated – Sigma Aldrich
Cellulose	Microcrystalline – Sigma Aldrich
FeCl_2_·4H_2_O	Sigma Aldrich (98%)
FeCl_3_·6H_2_O	Sigma Aldrich (≥98%)
Ammonia	Merck (25%)
Sodium hydroxide	Merck, 97%
CuCl_2_·2H_2_O	Sigma Aldrich (≥99%)
Ethanol	Sigma Aldrich – 97%
Sodium borohydride	Sigma Aldrich (≥96%)
FT-IR analysis	Shimadzu IR-470 spectrometer
EDX analysis	Numerix DXP-X10P
SEM analysis	Sigma-Zeiss microscope
TEM analysis	Philips Cm 12 Instrument
VSM analysis	Lakeshore 7407
TGA/DTA analysis	STA504 device
XRD analysis	JEOL JDX-8030 (30 kV, 20 mA)
BET analysis	Micromeritics ASAP 2010
XPS analysis	K-Alpha+
ICP-OES analysis	DV 5300
ICP-MS analysis	ELAN 6100 DRC-e
NMR analysis	Varian Unity Inova 500 MHz
Ultrasound cleaning bath	KQ-250 DE (40 kHz, 250 W)
Melting point measurement	Electrothermal 9100, made in UK

### 3.2. Preparation methods

#### 3.2.1. Preparation of magnetic Fe_3_O_4_@VPMP micro-plates

First, the purchased pumice was placed in a milling bowl and ground by ball-milling (20 Hz) for two hours. The ground pumice was then poured into a crucible and placed in a furnace until its temperature reached 400 °C within an hour, and then the temperature slowly reached the ambient temperature as it was inside the furnace and without any temperature shock. The pumice was then removed from the furnace and washed several times with HCl (1 M). Then, 2.2 g of the prepared pumice was dispersed with 5.0 mmol of FeCl_3_, 5.0 mmol of FeCl_2_ and 50.0 mL of deionized water in a 100.0 mL balloon for 30 minutes at 45 °C *via* ultrasonication using a cleaner bath. It was then placed on a stirrer at 85 °C and stirred vigorously under N_2_ atmosphere for two hours. After this time, 7.5 mL of 25% ammonia was added dropwise in one hour. After this process, ultimately, the product was magnetically separated from the water, washed with deionized water several times, and then dried in an oven.

#### 3.2.2. Preparation of the Fe_3_O_4_@VPMP/CLS composite

At this stage, 0.11 g of cellulose powder was initially dispersed in 20.0 mL of deionized water at room temperature. Then, 1.0 g of Fe_3_O_4_@VPMP particles were added with vigorous stirring to the prepared cellulose solution. After 6 hours, the Fe_3_O_4_@VPMP/CLS composite was magnetically separated and washed several times with deionized water.

#### 3.2.3. Preparation of the Fe_3_O_4_@VPMP/CLS–Cu(ii) composite

At this stage, in order to prepare the Fe_3_O_4_@VPMP/CLS–Cu(ii) composite, initially, 75.0 mL of 0.04 M solution of CuCl_2_·3H_2_O was prepared. Then, in another flask, Fe_3_O_4_@VPMP/CLS particles (1.0 g) were dispersed in deionized water *via* ultrasonication, and the as-prepared solution of NaOH (0.5 M, 50.0 mL) was added dropwise during the ultrasonication at room temperature. Afterward, the pre-prepared solution of CuCl_2_·3H_2_O was slowly added to the flask of solution containing Fe_3_O_4_@VPMP/CLS particles and stirred vigorously. After 6 hours, the Fe_3_O_4_@VPMP/CLS–Cu(ii) composite was magnetically separated and washed several times with deionized water.

### 3.3. A general procedure for reduction of nitroarenes with the Fe_3_O_4_@VPMP/CLS–Cu(0) system

To a round-bottom flask (25 mL) containing deionized water (5 mL), nitrobenzene (1 mmol, 0.123 g) and Fe_3_O_4_@VPMP/CLS–Cu(ii) (0.05 g) were added. Then, the pH of the mixture was tuned to *ca.* 8.0 by addition of K_2_CO_3_ (0.04 g). Next, NaBH_4_ (2 mmol, 0.075 g) was added to the mixture, and the resulting mixture was stirred at 70 °C. After completion of the reaction, the catalyst was separated by an external magnet and the product was extracted with EtOAc. The organic layer was dried over anhydrous Na_2_SO_4_. Evaporation of the solvent under reduced pressure afforded the pure aniline in 98% yield.

### 3.4. Large scale experiment on the reduction of nitrobenzene by the Fe_3_O_4_@VPMP/CLS–Cu catalytic system

To evaluate the efficiency of the prepared Fe_3_O_4_@VPMP/CLS–Cu catalytic system in industrial applications, a gram scale model experiment was carried out. For this purpose, in a round bottom flask (500 mL), 40 mmol (4.9 g) of nitrobenzene was dissolved in 160 mL of water. Then, the pH of the mixture was tuned to *ca.* 8.0 by addition of K_2_CO_3_ (1.6 g), and the temperature of the flask was controlled with an ice bath. In a separate flask (100 mL), 2.0 g of Fe_3_O_4_@VPMP/CLS–Cu powder was well dispersed in 40 mL of water *via* ultrasonication by a cleaner bath (50 KHz, 100 W L^−1^) and finally added to the main flask (500 mL) containing the nitrobenzene solution. Then, NaBH_4_ (80 mmol, 3.0 g) was added to the mixture, and the resulting mixture was stirred in an ice bath. At this stage, the reaction temperature was maintained at around 20 °C. After completion of the reaction, the magnetic particles were separated with an external magnet several times. Next, the residue was divided into five 40 mL portions, and liquid–liquid extraction was performed using EtOAc (20 mL) (three times for each portion). Finally, the organic phase was dried over anhydrous Na_2_SO_4_ (5.0 g), and the solvent was evaporated under reduced pressure (pure aniline in 98% yield).

## 4. Conclusion

In this study, a magnetic catalytic system using natural-based materials was presented that has the ability to reduce various derivatives of nitrobenzene (NB) in a very short time of 8 minutes. This efficient catalytic system was characterized by FT-IR spectroscopy, SEM, EDX spectroscopy, XRD spectroscopy, TGA, and VSM. This catalytic system has many advantages, such as (1) convenient separation, which is due to the superparamagnetic behavior of the nanoscale catalytic system, which was also proven by VSM analysis; (2) due to the high porosity of the pumice according to SEM, this catalytic system is able to provide a large active surface area; and (3) the use of low amounts of the heterogeneous catalyst (0.05 g) and many other advantages can introduce this composite as a suitable candidate for scaling up and industrial applications.

## Author contributions

R. T. L. designed the project and led the hypothesis. M. S. and F. S. Q. carried out the experimental sections and provided the required analyses. R. T. L. and M. S. prepared the first draft of manuscript. All the authors helped in interpreting the obtained results, writing, editing and revising the manuscript. A. M. and A. E. S. managed and supervised all the work sections.

## Conflicts of interest

The authors listed in this article have no conflict of interests.

## Supplementary Material

RA-011-D1RA03538J-s001
