# Expression of concern: Highly porous copper-supported magnetic nanocatalysts: made of volcanic pumice textured by cellulose and applied for the reduction of nitrobenzene derivatives

**DOI:** 10.1039/d4ra90026j

**Published:** 2024-03-25

**Authors:** Reza Taheri-Ledari, Mahdi Saeidirad, Fateme Sadat Qazi, Atefeh Fazeli, Ali Maleki, Ahmed Esmail Shalan

**Affiliations:** a Catalysts and Organic Synthesis Research Laboratory, Department of Chemistry, Iran University of Science and Technology Tehran 16846-13114 Iran maleki@iust.ac.ir; b BC Materials, Basque Center for Materials, Applications and Nanostructures, Martina Casiano, UPV/EHU Science Park Barrio Sarriena s/n Leioa 48940 Spain a.shalan133@gmail.com ahmed.shalan@bcmaterials.net; c Central Metallurgical Research and Development Institute (CMRDI) P. O. Box 87, Helwan Cairo 11421 Egypt

## Abstract

Expression of concern for ‘Highly porous copper-supported magnetic nanocatalysts: made of volcanic pumice textured by cellulose and applied for the reduction of nitrobenzene derivatives’ by Reza Taheri-Ledari *et al.*, *RSC Adv.*, 2021, **11**, 25284–25295, https://doi.org/10.1039/D1RA03538J.

The Royal Society of Chemistry is publishing this expression of concern in order to alert readers that concerns have been raised regarding the reliability of the SEM-energy mapping data in Fig. 3g. An investigation is underway, and an expression of concern will continue to be associated with the article until a final outcome is reached.

Laura Fisher

19th March 2024

Executive Editor, *RSC Advances*

## Supplementary Material

