# Supplementary material for: Highly porous copper-supported magnetic nanocatalysts: made of volcanic pumice textured by cellulose and applied for the reduction of nitrobenzene derivatives
Source: RSC Adv. 2021 Jul 21;11(41):25284–95. doi: 10.1039/d1ra03538j (PMC9036973; doi:10.1039/d1ra03538j)
Supplement: RA-011-D1RA03538J-s001 [file RA-011-D1RA03538J-s001.pdf]

Electronic Supplementary Material (ESI) for RSC Advances.
This journal is © The Royal Society of Chemistry 2021

Supplementary Information

Highly porous copper-supported magnetic nanocatalyst: Made of volcanic pumice textured by cellulose, applied for reduction of nitrobenzene derivatives

Reza Taheri-Ledari[1], Mahdi Saeidirad[1], Fateme Sadat Qazi[1], Atefeh Fazeli[1], Ali Maleki*[,1], Ahmed Esmail Shalan*[,2,3,+]

[1] *Catalysts and Organic Synthesis Research Laboratory, Department of Chemistry, Iran University of Science and Technology (IUST), Tehran 16846-13114, Iran.*

[2] *BCMaterials, Basque Center for Materials, Applications and Nanostructures, Martina Casiano, UPV/EHU Science Park, Barrio Sarriena s/n, Leioa 48940, Spain.*

[3] *Central Metallurgical Research and Development Institute (CMRDI), P.O. Box 87, Helwan, Cairo 11421, Egypt.*

**Corresponding authors. (A. M) Tel.: +98 21 77240640-50; fax: +98 21 73021584. E-mail address: maleki@iust.ac.ir; (A. E. S.) E-mail: ahmed.shalan@bcmaterials.net; a.shalan133@gmail.com*

[+] *Currently on leave from CMRDI.*

Author's ORCIDs:

Reza Taheri-Ledari: https://orcid.org/0000-0002-6511-9411

Ali Maleki: https://orcid.org/0000-0001-5490-3350

Ahmed Esmail Shalan: https://orcid.org/0000-0002-3424-1609

Table of content

Content	Page
**Figure S1.** ^{1}H-NMR spectra and spectral data of aniline	S3
**Figure S2.** ^{13}C-NMR spectra and spectral data of aniline	S4
**Figure S3.** ^{1}H-NMR spectra and spectral data of benzene-1,4-diamine	S5
**Figure S4.** ^{13}C-NMR spectra and spectral data of benzene-1,4-diamine	S6
**Figure S5.** ^{1}H-NMR spectra and spectral data of 2-amino-4-chloro-phenol	S7
**Figure S6.** ^{13}C-NMR spectra and spectral data of 2-amino-4-chloro-phenol	S8
**Figure S7.** ^{1}H-NMR spectra and spectral data of 2-aminophenol	S9
**Figure S8.** ^{13}C-NMR spectra and spectral data of 2-aminophenol	S10
**Figure S9.** ^{1}H-NMR spectra and spectral data of 4-amino-benzonic acid	S11
**Figure S10.** ^{13}C-NMR spectra and spectral data of 4-amino-benzonic acid	S12
**Figure S11.** ^{1}H-NMR spectra and spectral data of 4-amino-phenol	S13
**Figure S12.** ^{13}C-NMR spectra and spectral data of 4-amino-phenol	S14
**Figure S13.** ^{1}H-NMR spectra and spectral data of benzene-1,3-diamine	S15
**Figure S14.** ^{13}C-NMR spectra and spectral data of benzene-1,3-diamine	S16
**Figure S15.** ^{1}H-NMR spectra and spectral data of 2-Naphthylamine	S17
**Figure S16.** ^{13}C-NMR spectra and spectral data of 2-Naphthylamine	S18
**Figure S17.** ^{1}H-NMR spectra and spectral data of p-toluidine	S19
**Figure S18.** ^{13}C-NMR spectra and spectral data of p-toluidine	S20
**Figure S19.** ^{1}H-NMR spectra and spectral data of 4-chloroaniline	S21
**Figure S20.** ^{13}C-NMR spectra and spectral data of 4-chloroaniline	S22
**Table S1.** Results of ICP-OES analysis of Fe_3O_4@VPMP/CLS-Cu nanocomposite	S23
Estimation of the catalyst amount in mmol% based on ICP-OES results	S23
Calculation of turnover number (TON) and turnover frequency (TOF)	S24
Estimation of half-life of the reduction reaction of nitrobenzene and investigation of reusability of Fe_3O_4@VPMP/CLS-Cu catalytic system	S25
**Figure S21**. Calibration curves of nitrobenzene, and (b) UV-Vis absorbance spectra of the NB reaction mixture, under catalytic conditions in different times	S25
**Table S2**. The yields and half-life values obtained for the reduction reaction of nitrobenzene (NB), under catalytic conditions provided by Fe_3O_4@VPMP/CLS-Cu system during five times reuse	S26
**Table S3**. Results of ICP-MS analysis on copper ion exist in supernatants after separation of the magnetic particles, after running each recycling process (leaching test)	S26
**Table S4.** Results of ICP-OES analysis of the recovered Fe_3O_4@VPMP/CLS-Cu nanocomposite.	S27
**Figure S22**. The graph of the optimization stages of reduction reaction of nitrobenzene under various catalytic conditions (related to Table 1).	S28

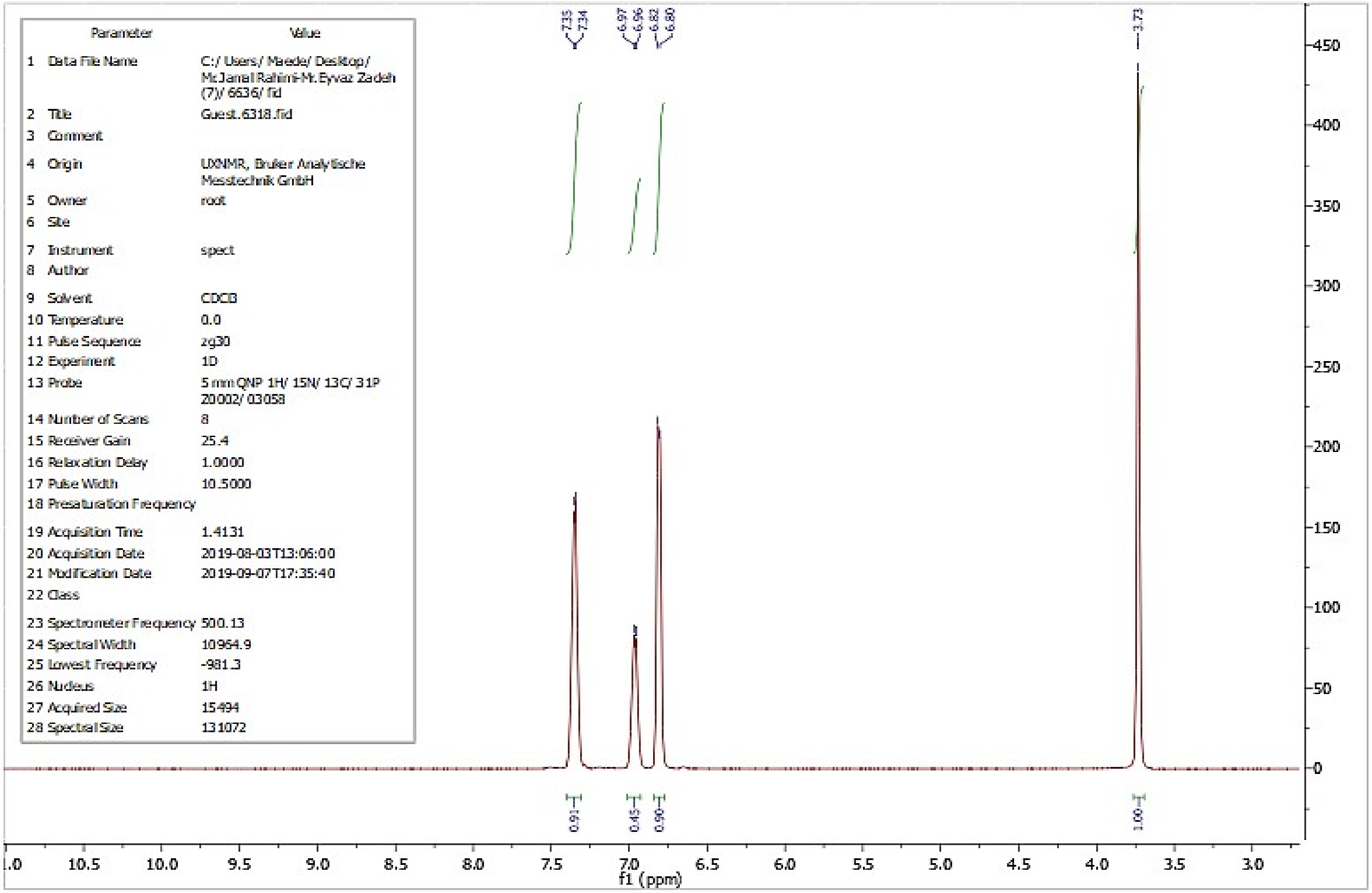

1. Aniline (**Table 2 – Entry 1)**

^{1}H NMR (500 MHz, $CDCl_3$): δ 3.73 (s, 2 H), 6.80-6.82 (d, J = 10 Hz, 2 H), 6.93-6.99(m, 1H), 7.34-7.35 (d, J = 5 Hz, 2 H).

Figure S1. ^{1}H-NMR spectrum and spectral data of aniline.

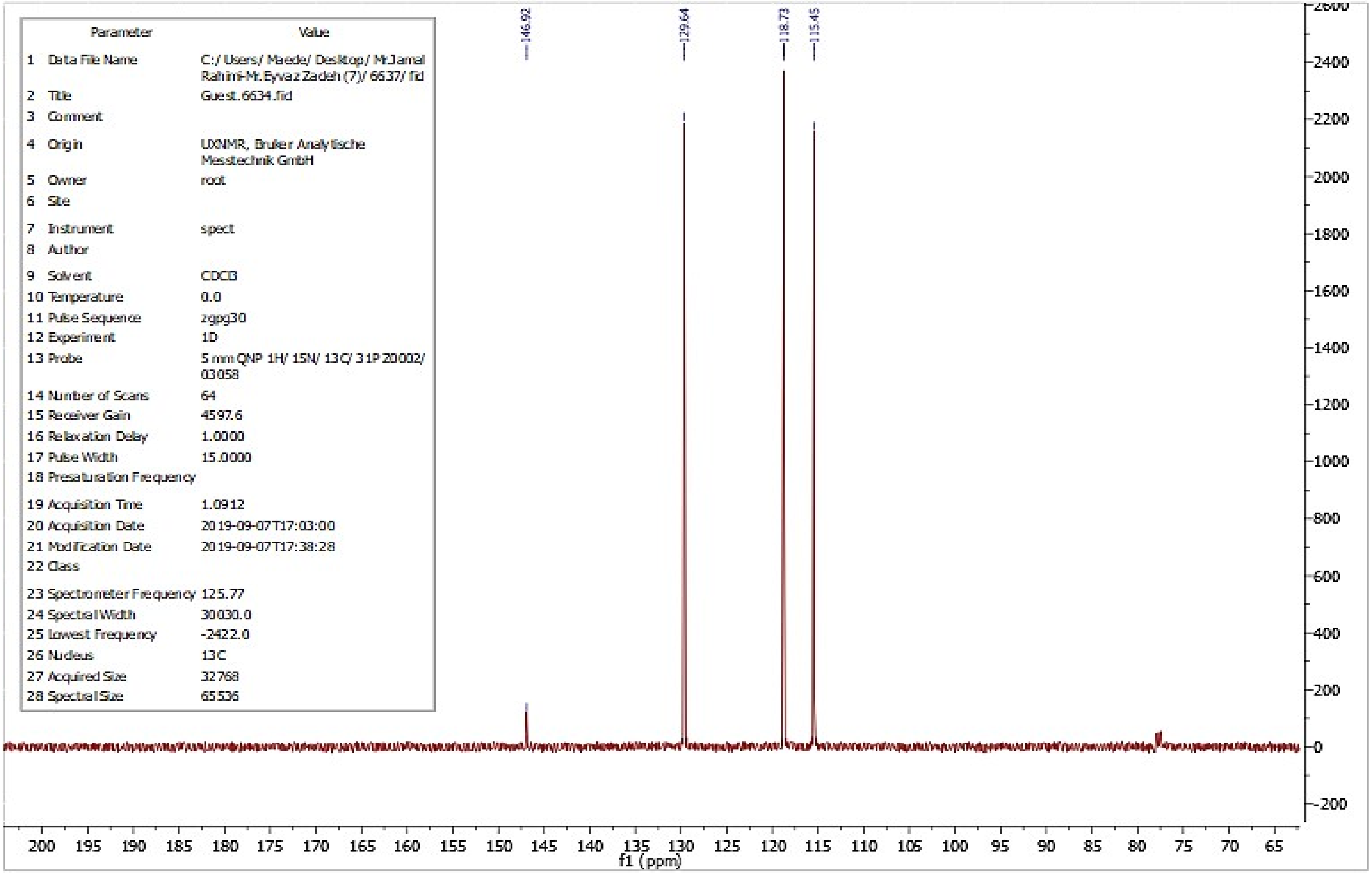

1. Aniline (**Table 2 – Entry 1)**

^{13}C NMR (500 MHz, $CDCl_3$): δ 146.92, 129.64, 118.73, 115.45.

Figure S2. ^{13}C NMR spectrum and spectral data of aniline.

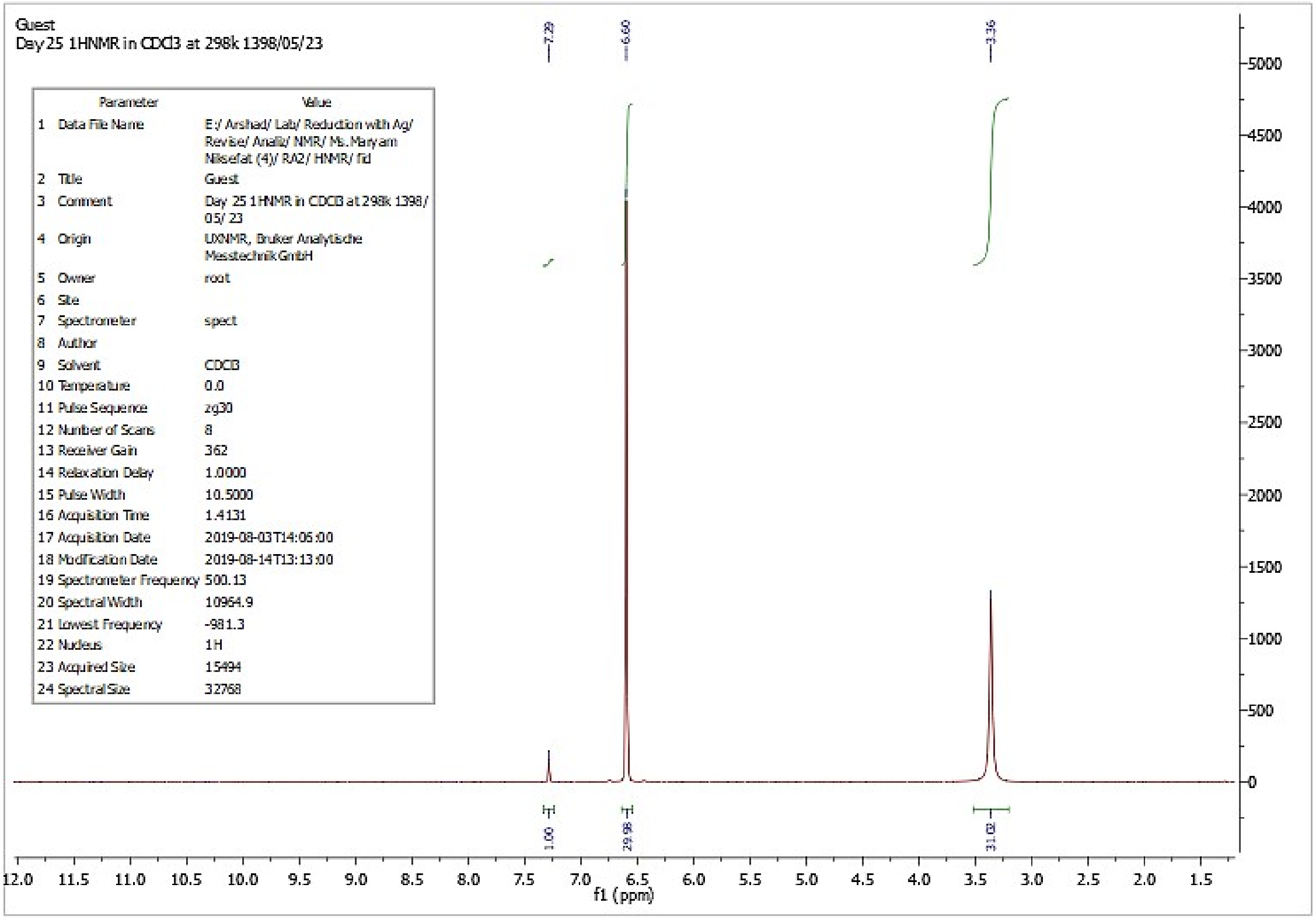

2. benzene-1,4-diamine (**Table2 – Entry 2)**

^{1}H NMR (500 MHz, $CDCl_3$): δ 6.59 (s, 4 H), 3.36 (s, 4 H).

Figure S3. ^{1}H-NMR spectrum and spectral data of benzene-1,4-diamine.

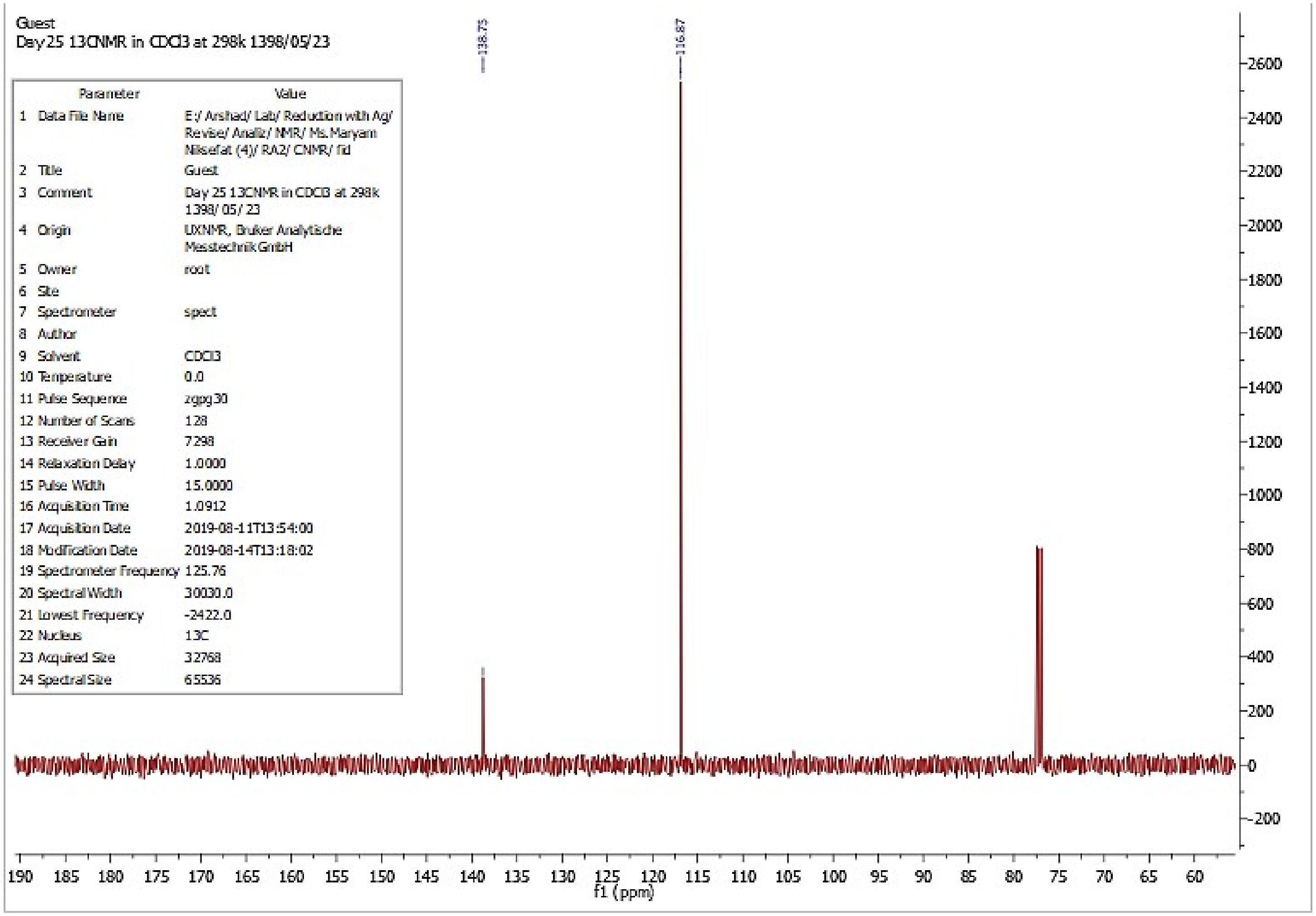

2. benzene-1,4-diamine (**Table2 – Entry 2)**

^{13}C NMR (500 MHz, $CDCl_3$): δ 138.75, 116.87.

Figure S4. ^{13}C-NMR spectra and spectral data of benzene-1,4-diamine.

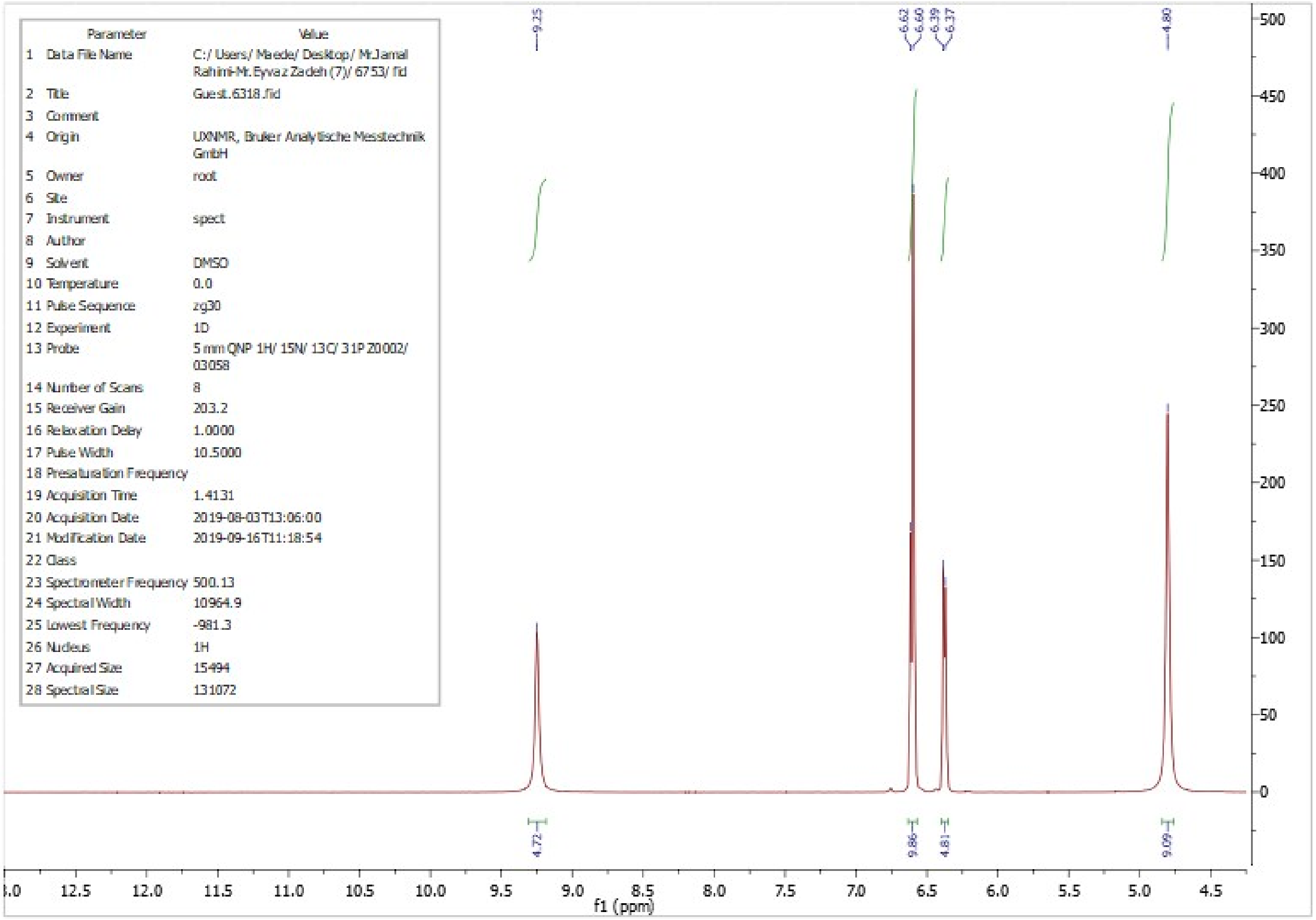

3. 2-amino-4-chloro-phenol **(Table 2 – Entry 3)**

^{1}H NMR (500 MHz, DMSO): δ 9.25 (s, 1H), 6.61 (m, 1H), 6.61 (m, 1H), 6.38 (m, 1H), 4.80 (s, 2H).

Figure S5. ^{1}H-NMR spectra and spectral data of 2-amino-4-chloro-phenol.

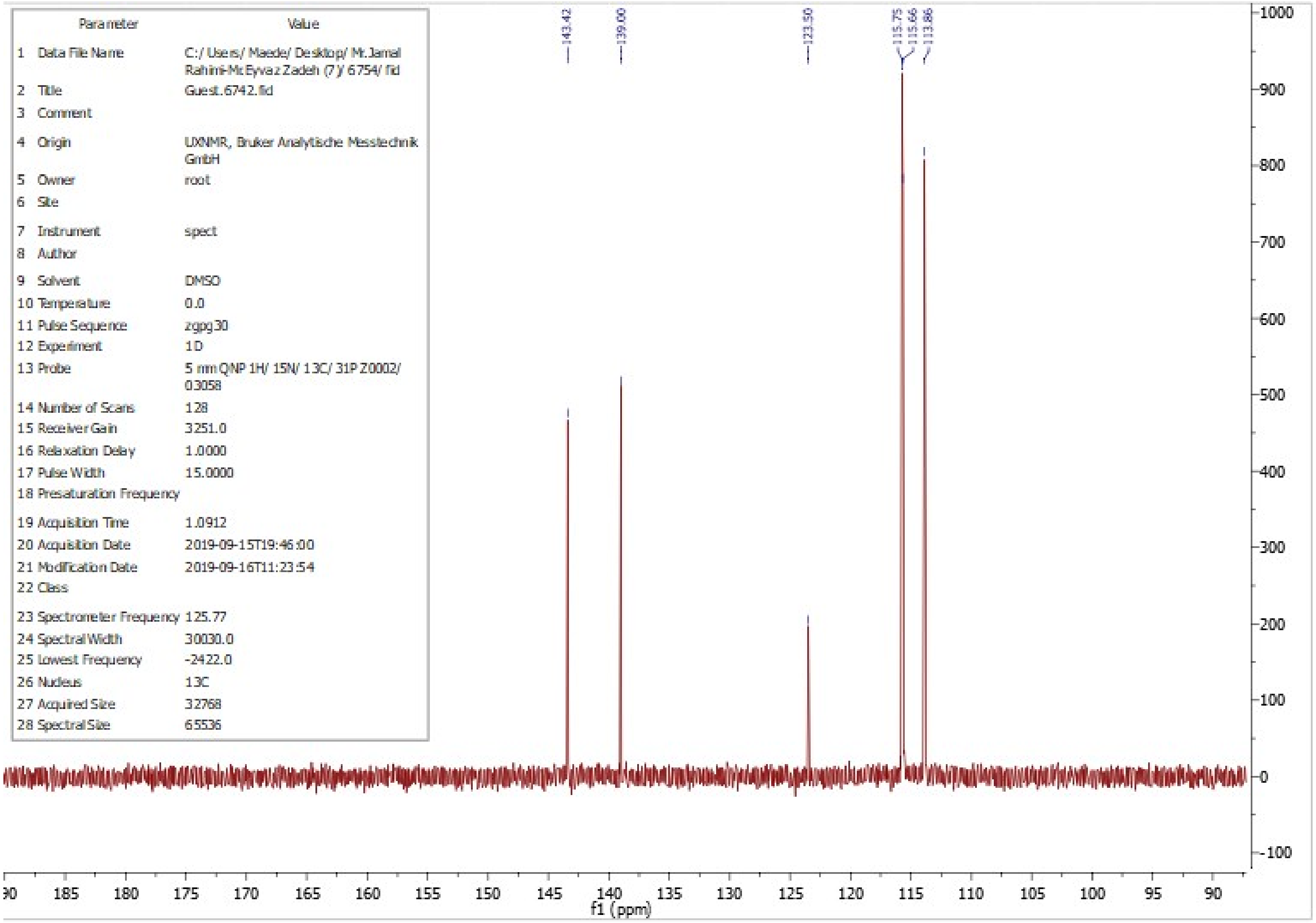

3. 2-amino-4-chloro-phenol (**Table 2 – Entry 3)**

^{13}C NMR (500 MHz, DMSO): δ 143.42, 139.00, 123.50, 115.75, 115.66, 113.86.

Figure S6. ^{13}C-NMR spectra and spectral data of 2-amino-4-chloro-phenol.

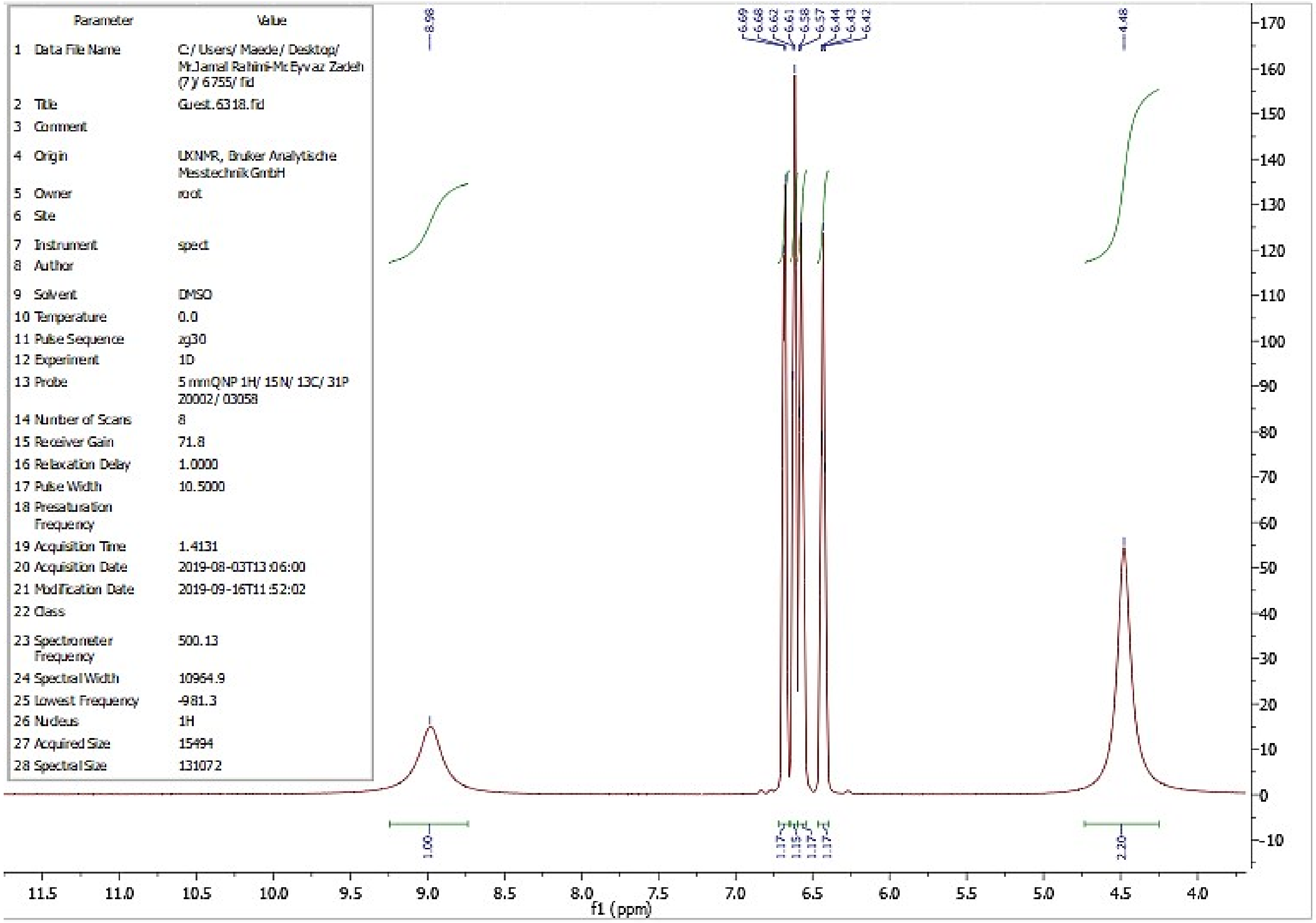

4. 2-aminophenol **(Table 2 – Entry 4)**

^{1}H NMR (500 MHz, DMSO): δ 8.98 (s, 1H), 6.68 (m, 1H), 6.61 (m, 1H), 6.57 (m, 1H), 6.43 (m, 1H), 4.48 (S, 2H).

Figure S7. ^{1}H-NMR spectra and spectral data of 2-aminophenol.

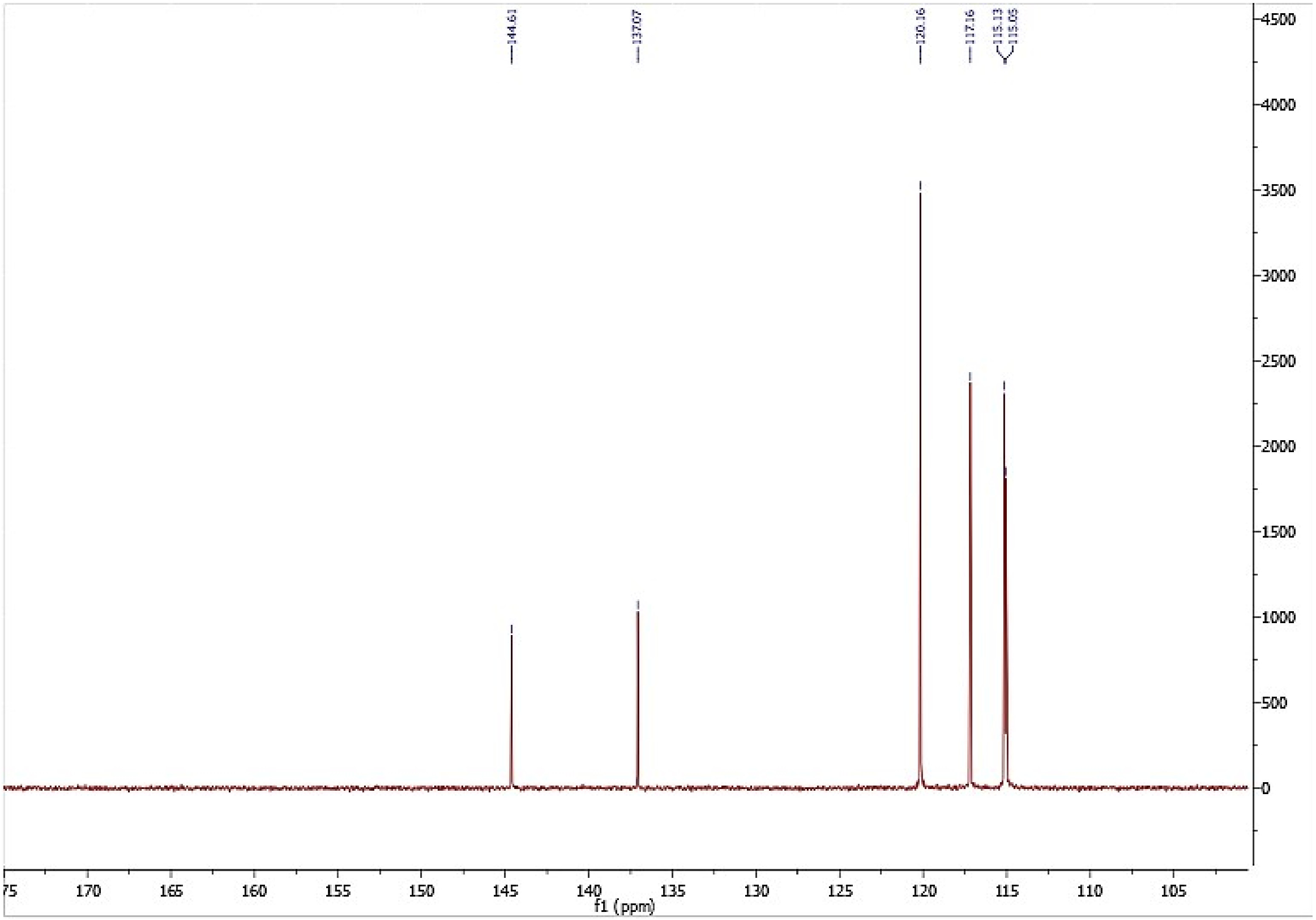

4. 2-aminophenol (**Table 2 – Entry 4)**

^{13}C NMR (500 MHz, DMSO): δ 144.51, 137.07, 120.16, 117.16, 115.13, 115.05.

Figure S8. ^{13}C-NMR spectra and spectral data of 2-aminophenol.

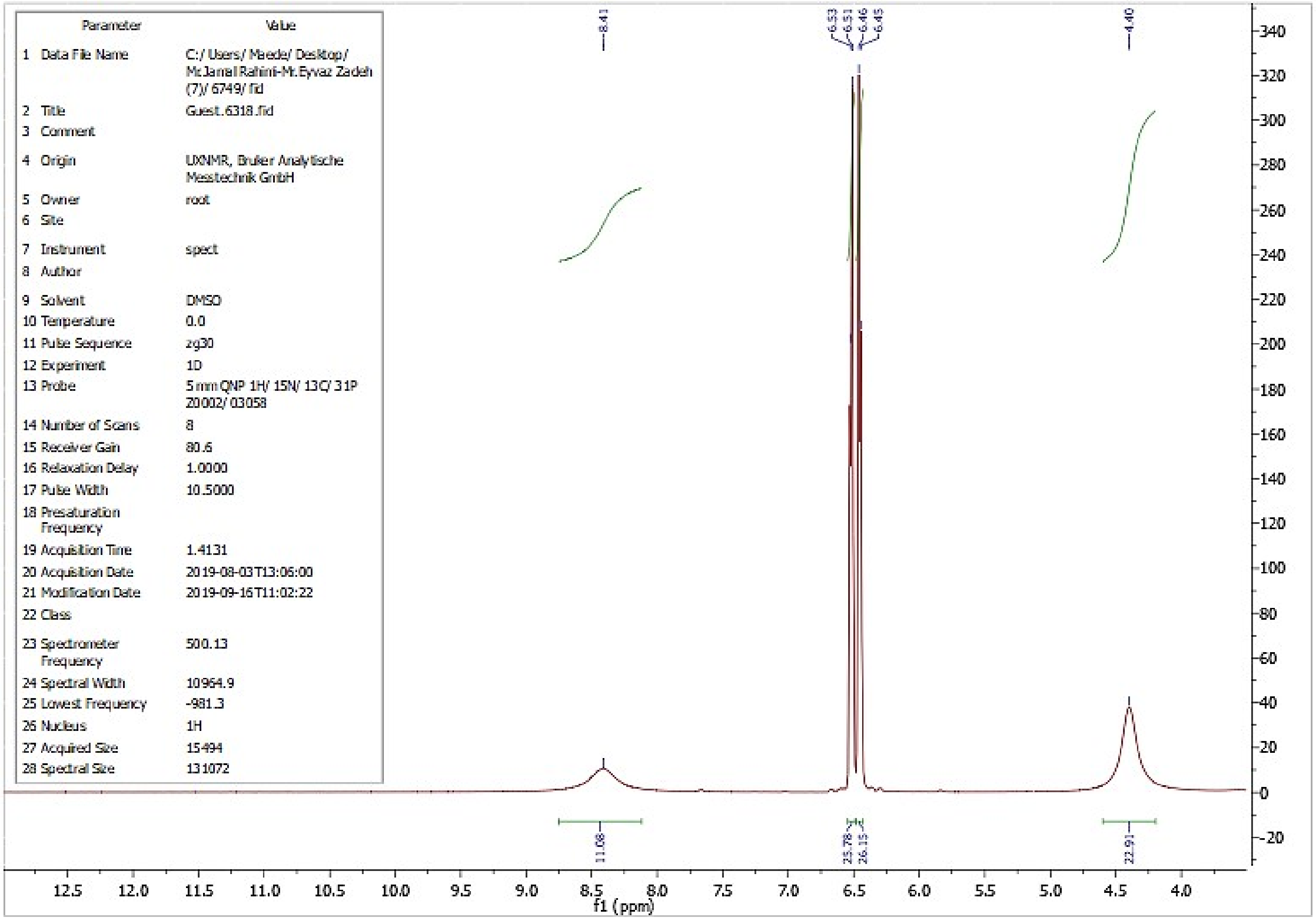

5. 4-amino-benzonic acid **(Table 2 – Entry 5)**

^{1}H NMR (500 MHz, DMSO): δ 8.41 (s, 1H), 6.53-6.51 (d, J = 10 Hz, 2H), 6.46-6.45 (d, J = 5 Hz, 2H), 4.40 (s, 2H).

Figure S9. ^{1}H-NMR spectra and spectral data of 4-amino-benzonic acid.

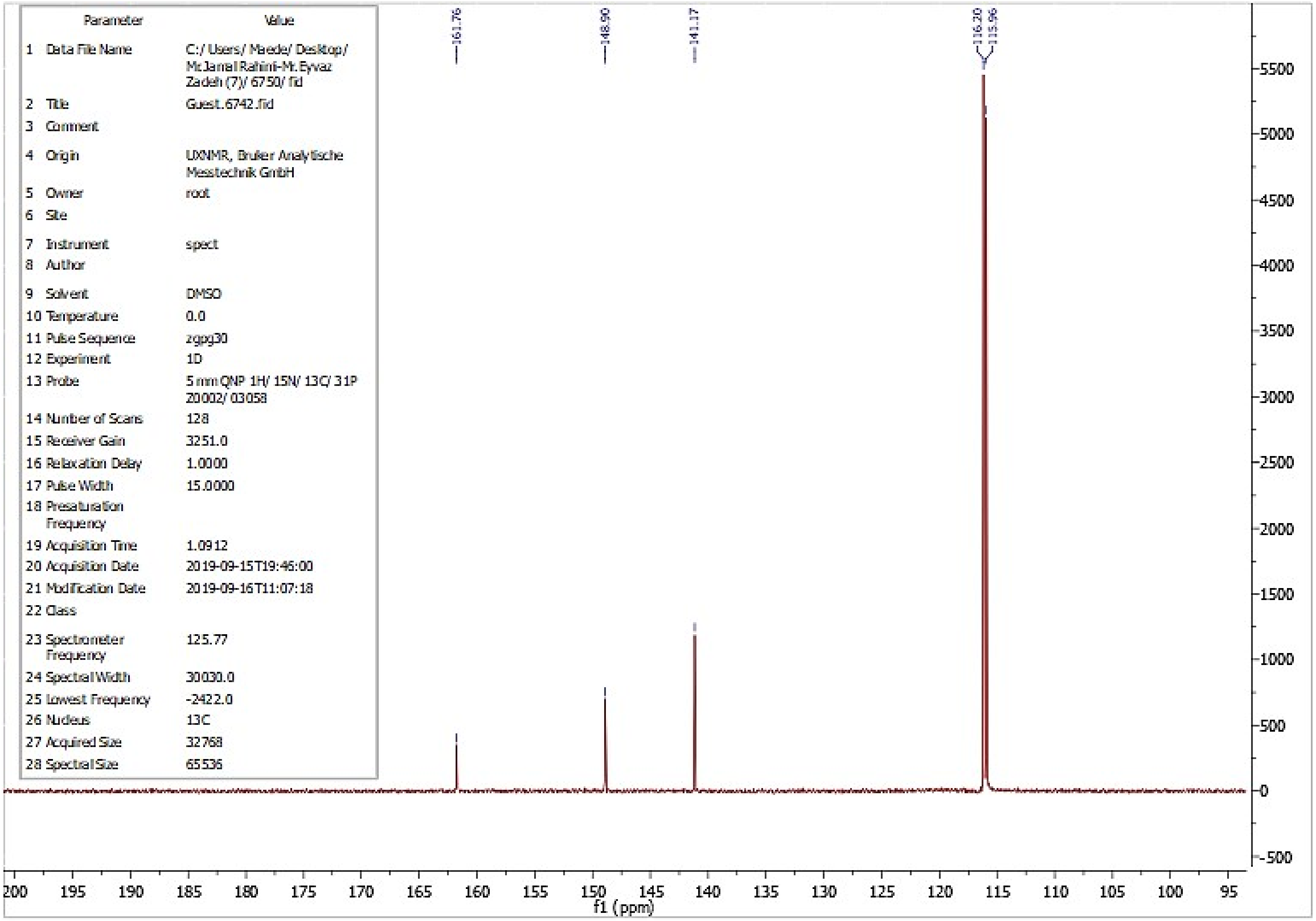

5. 4-amino-benzonic acid (**Table 2 – Entry 5)**

^{13}C NMR (500 MHz, DMSO): δ 161.76, 148.90, 141.17, 116.20, 115.96.

Figure S10. ^{13}C-NMR spectra and spectral data of 4-amino-benzonic acid.

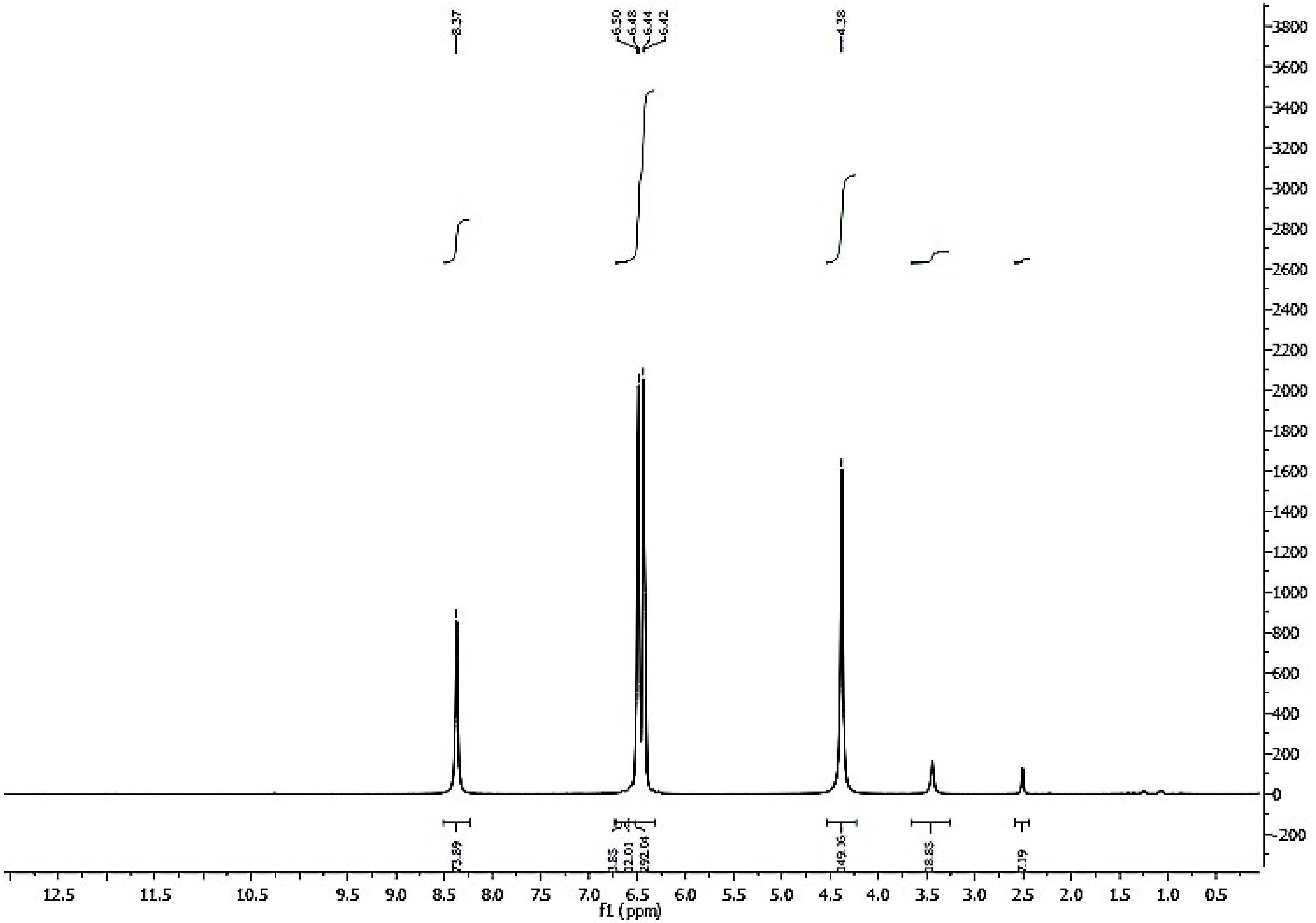

6. 4-amino-phenol **(Table 2 – Entry 6)**

^{1}H NMR (500 MHz, DMSO): δ (ppm) = 4.38 (2H, s, NH2), 6.42–6.44 (2H, d, J=10 Hz, H–Ar), 6.48–6.50 (2H, d, J=10 Hz, H–Ar), 8.37 (1H, s, OH).

Figure S11. ^{1}H-NMR spectra and spectral data of 4-amino-phenol.

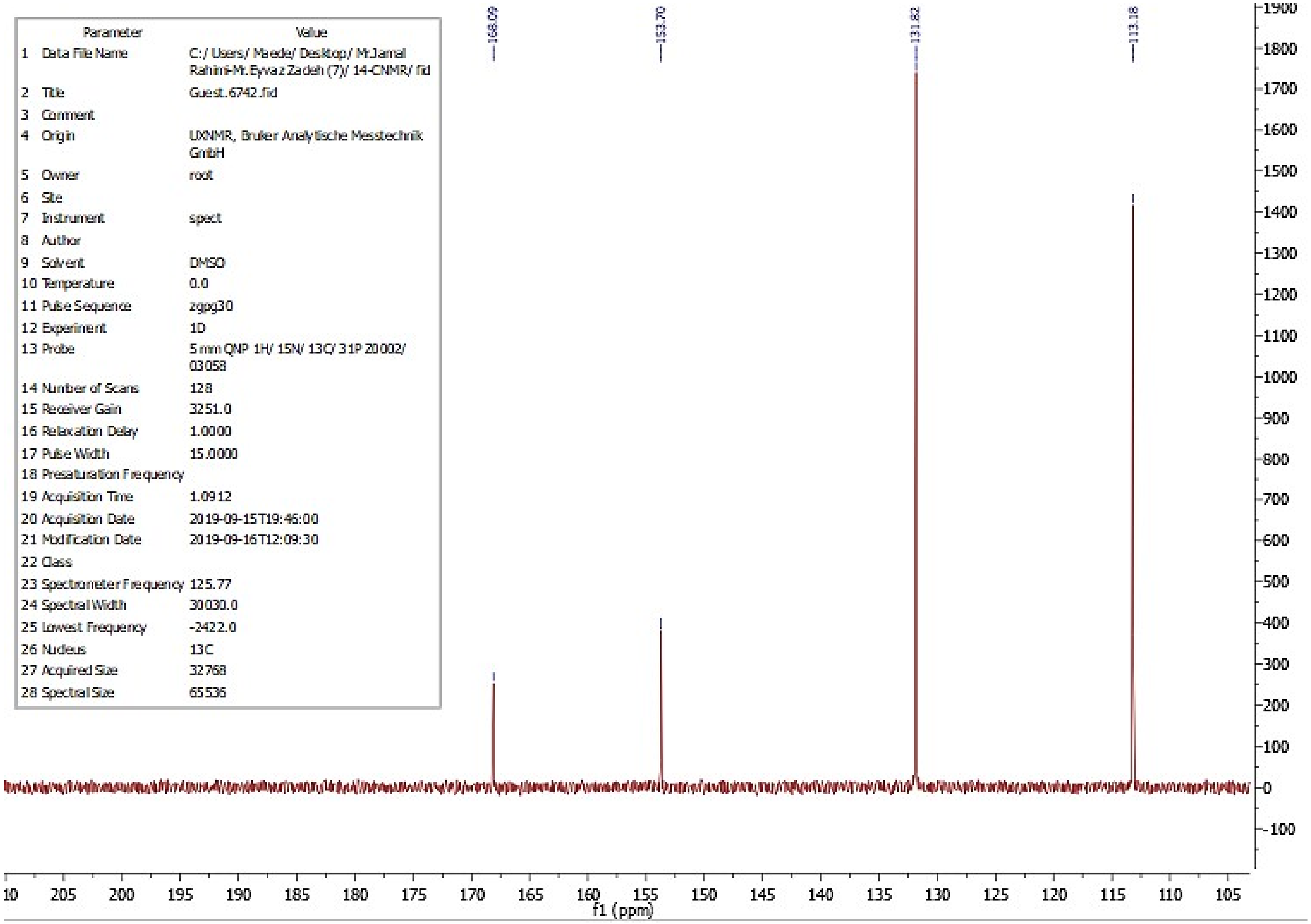

6. 4-amino-phenol (**Table 2 – Entry 6)**

^{13}C NMR (500 MHz, DMSO): δ 168.09, 153.70, 131.82, 113.18.

Figure S12. ^{13}C-NMR spectra and spectral data of 4-amino-phenol.

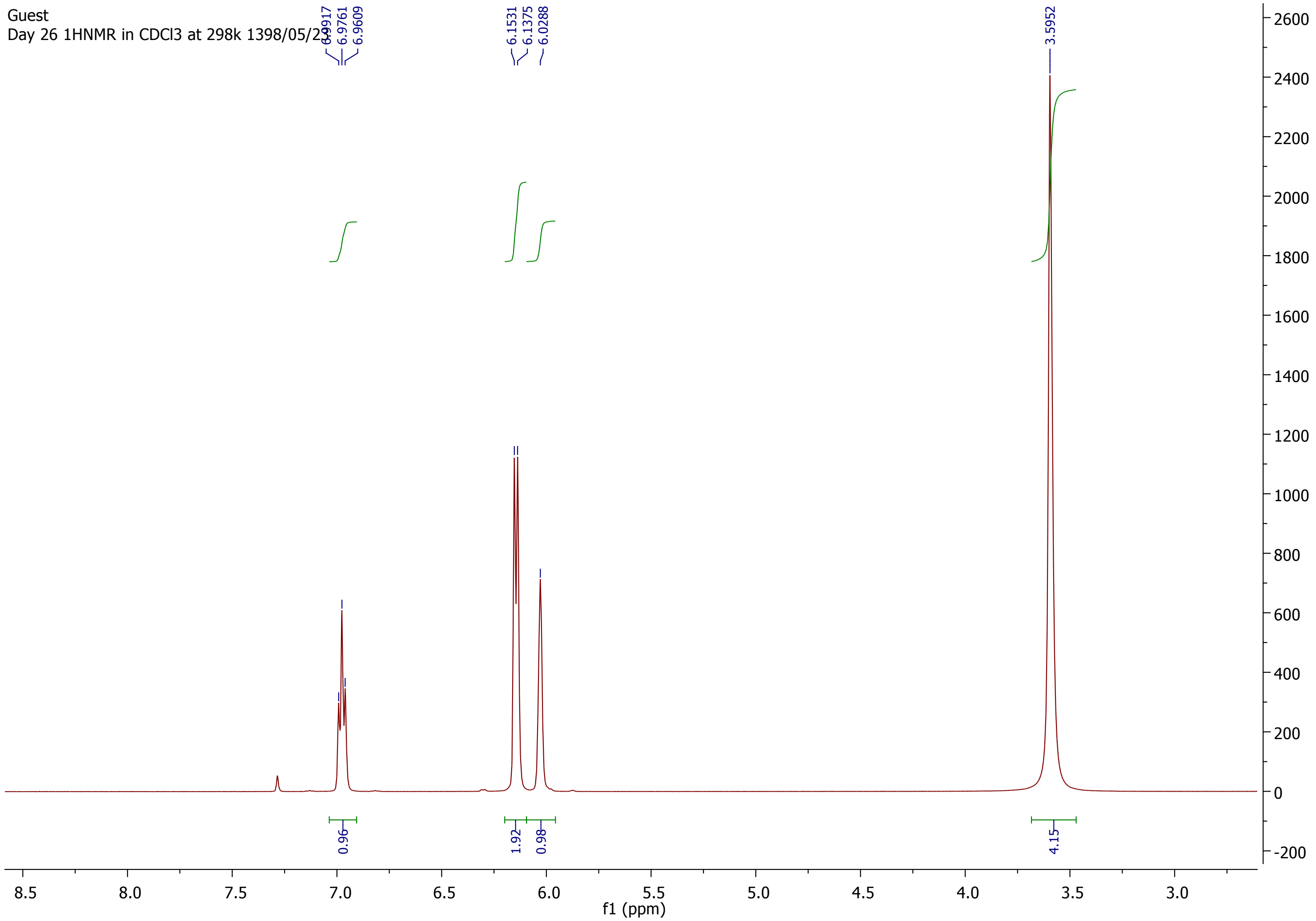

7. benzene-1,3-diamine **(Table 2 – Entry 7)**

^{1}H NMR (500 MHz, CDCl3): δ 6.96-6.99 (m, 1 H), 6.15–6.13 (d, J = 7.8 Hz, 2 H), 6.03 (s, 1 H), 3.59 (s, 4 H).

Figure S13. ^{1}H-NMR spectra and spectral data of benzene-1,3-diamine.

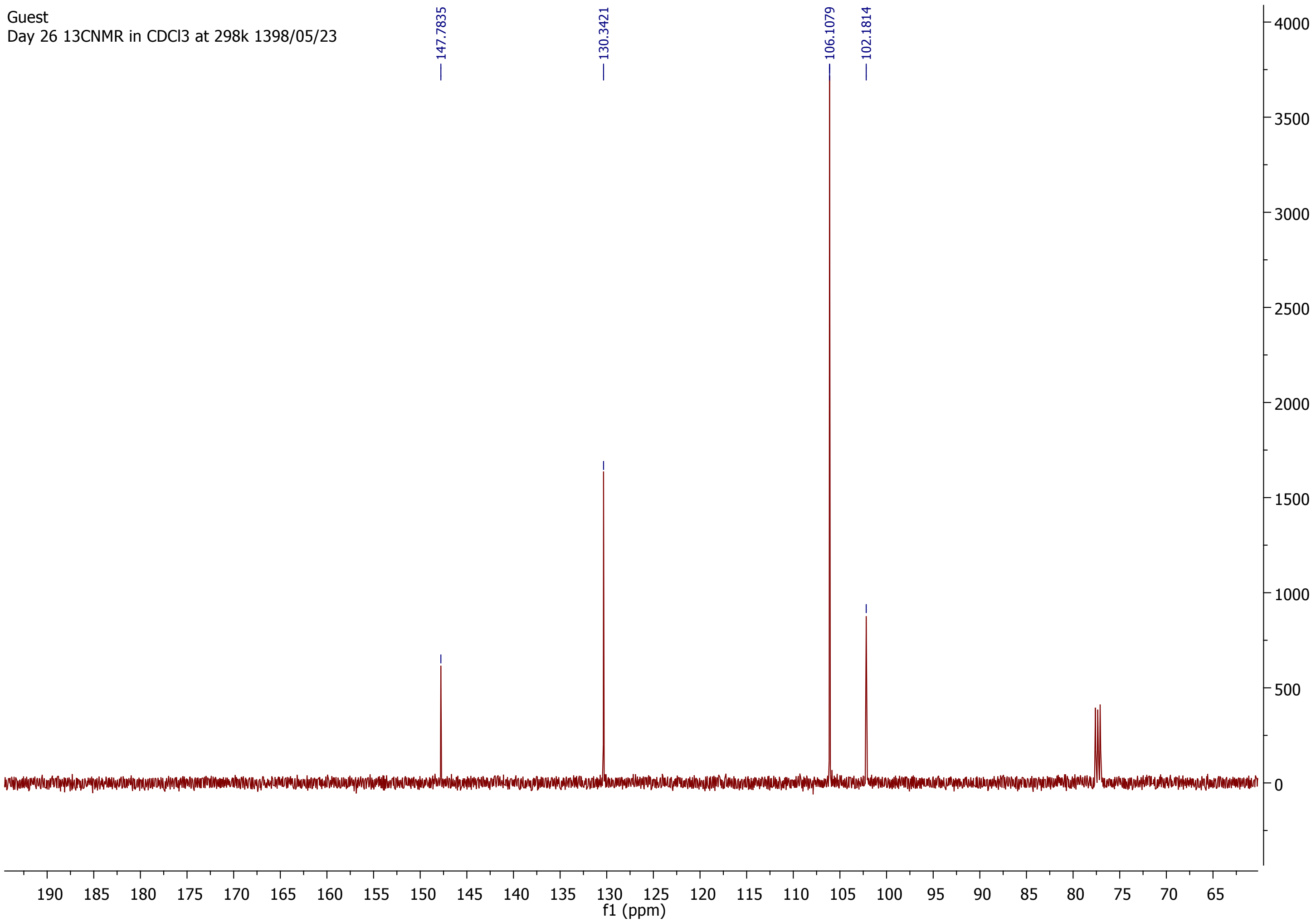

7. benzene-1,3-diamine (**Table 2 – Entry 7)**

^{13}C NMR (500 MHz, $CDCl_3$): δ 147.78, 130.34, 106.25, 102.18.

Figure S14. ^{13}C-NMR spectra and spectral data of benzene-1,3-diamine.

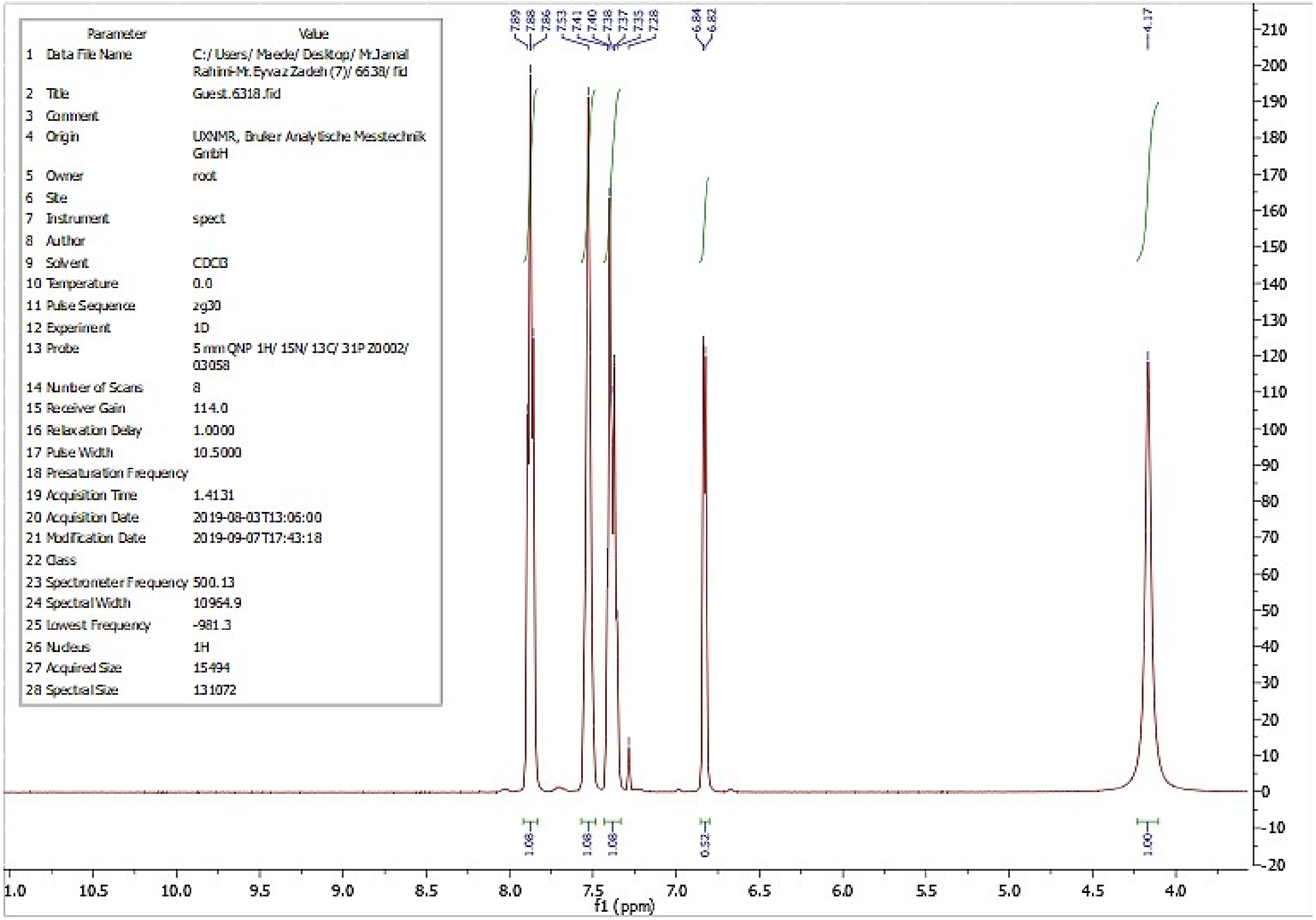

8. 2-Naphthylamine **(Table 2 – Entry 8)**

^{1}H-NMR (500 MHz, CDCl3): δ = 7.88 (m, 2H), 7.53 (m, 2H), 7.38 (m, 2H), 6.83 (m, 1H), 4.17 (s, 2H).

Figure S15. ^{1}H-NMR spectra and spectral data of 2-Naphthylamine.

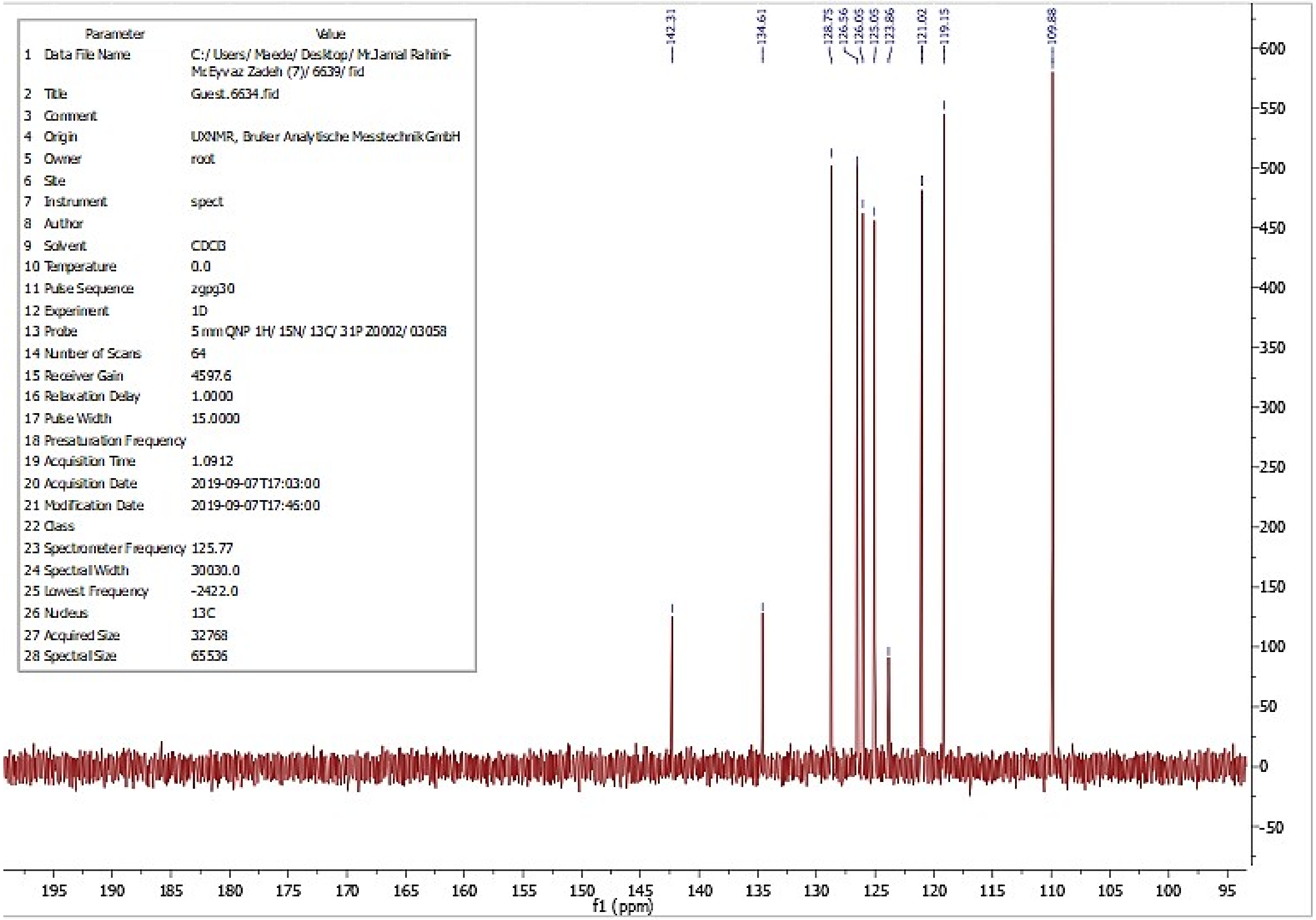

8. 2-Naphthylamine (**Table 2 – Entry 8)**

^{13}C-NMR (500 MHz, $CDCl_3$): δ = 142.31, 134.61, 128.75, 126.56, 126.05, 125.05, 123.86, 121.02, 119.15, 109.88.

Figure S16. ^{13}C-NMR spectra and spectral data of 2-Naphthylamine.

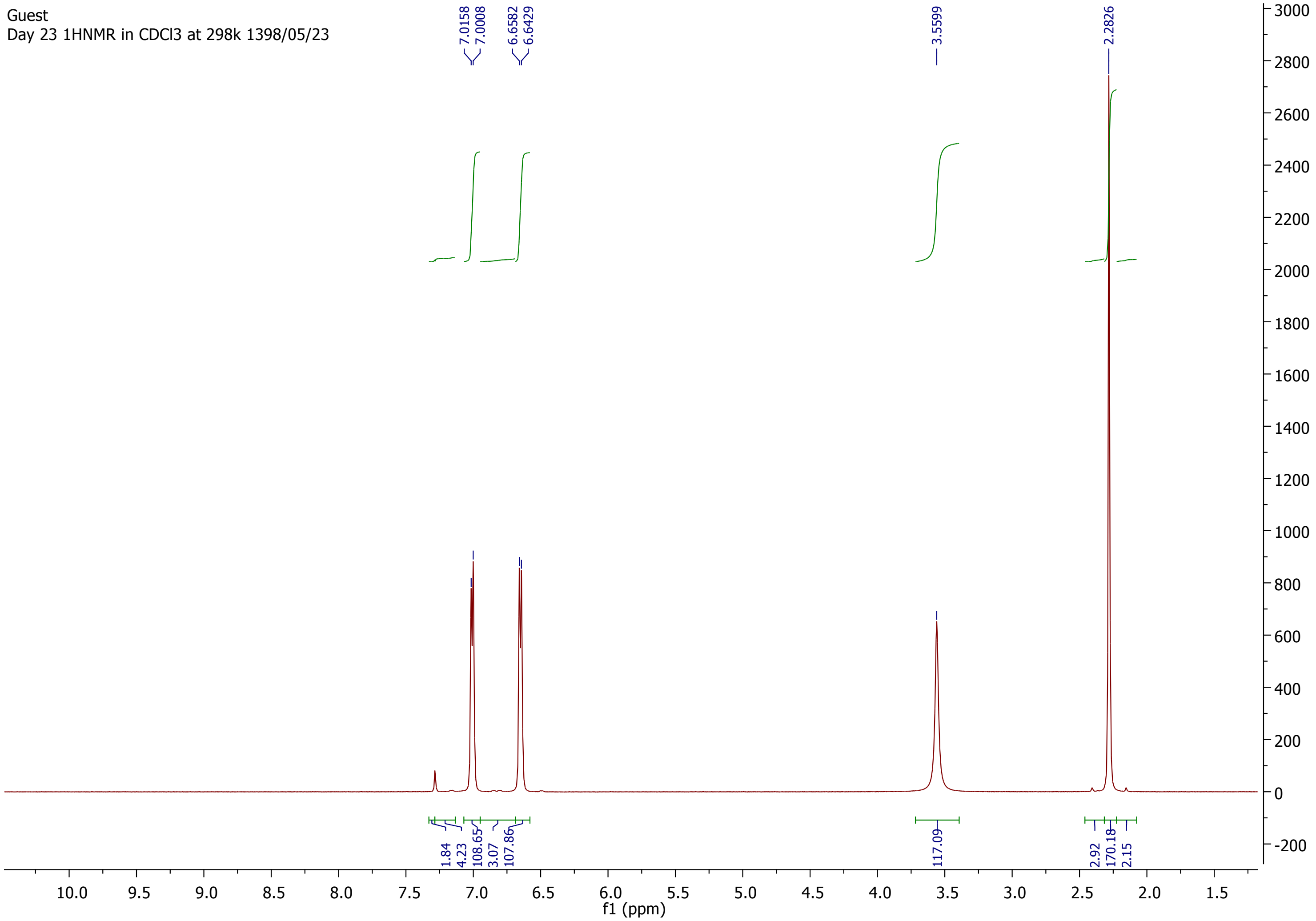

9. p-toluidine **(Table 2 – Entry 9)**

^{1}H NMR (500 MHz, CDCl3): δ 2.28 (s, 3H), 3.56 (s, 2H), 6.64-6.66 (d, J = 10 Hz, 2H), 7.00-7.02 (d, J = 10 Hz, 2H).

Figure S17. ^{1}H-NMR spectra and spectral data of p-toluidine.

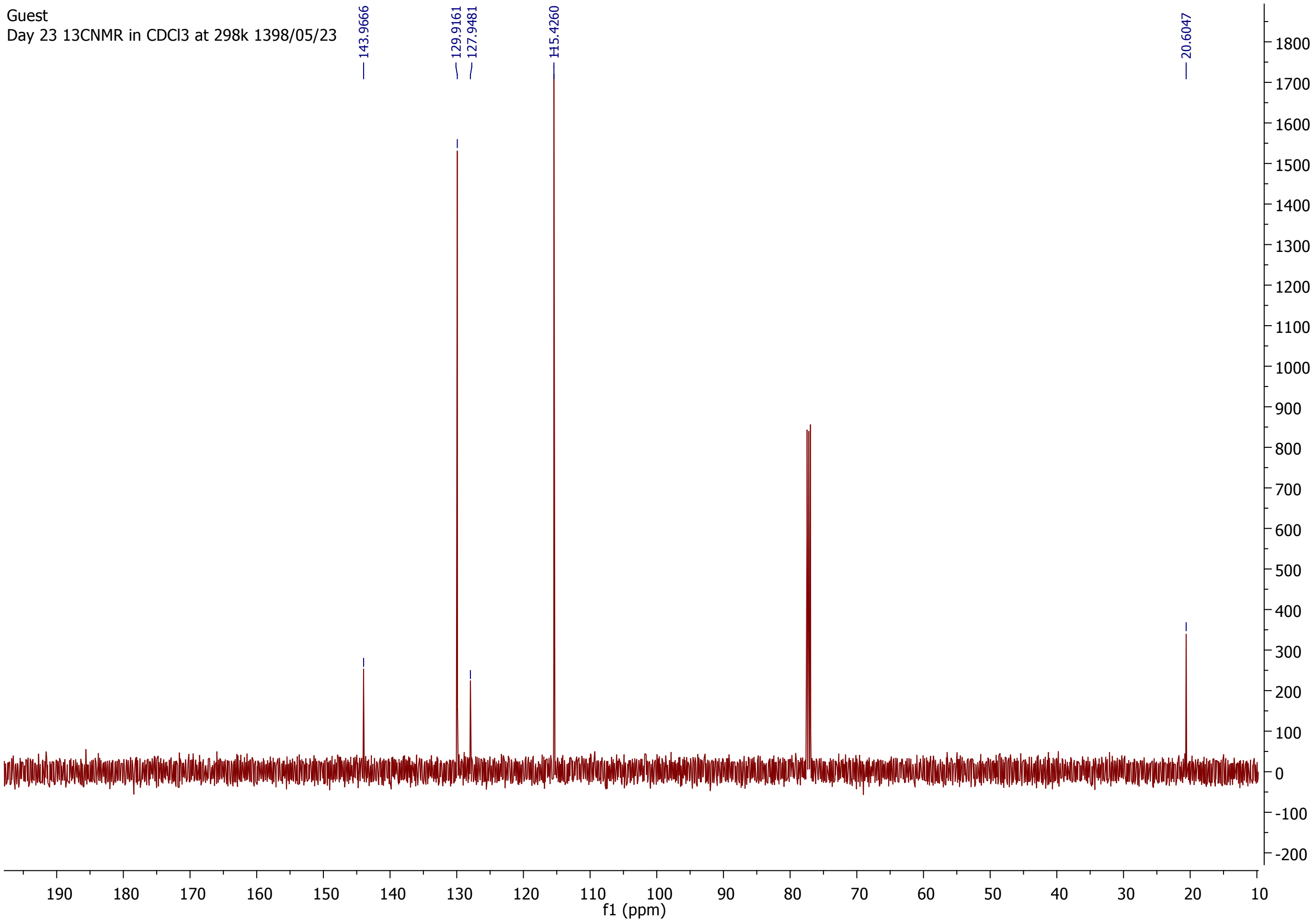

9. p-toluidine (**Table 2 – Entry 9)**

^{13}C NMR (500 MHz, $CDCl_3$) δ 20.60, 115.43, 127.96, 129.92, 143.97.

Figure S18. ^{13}C-NMR spectra and spectral data of p-toluidine.

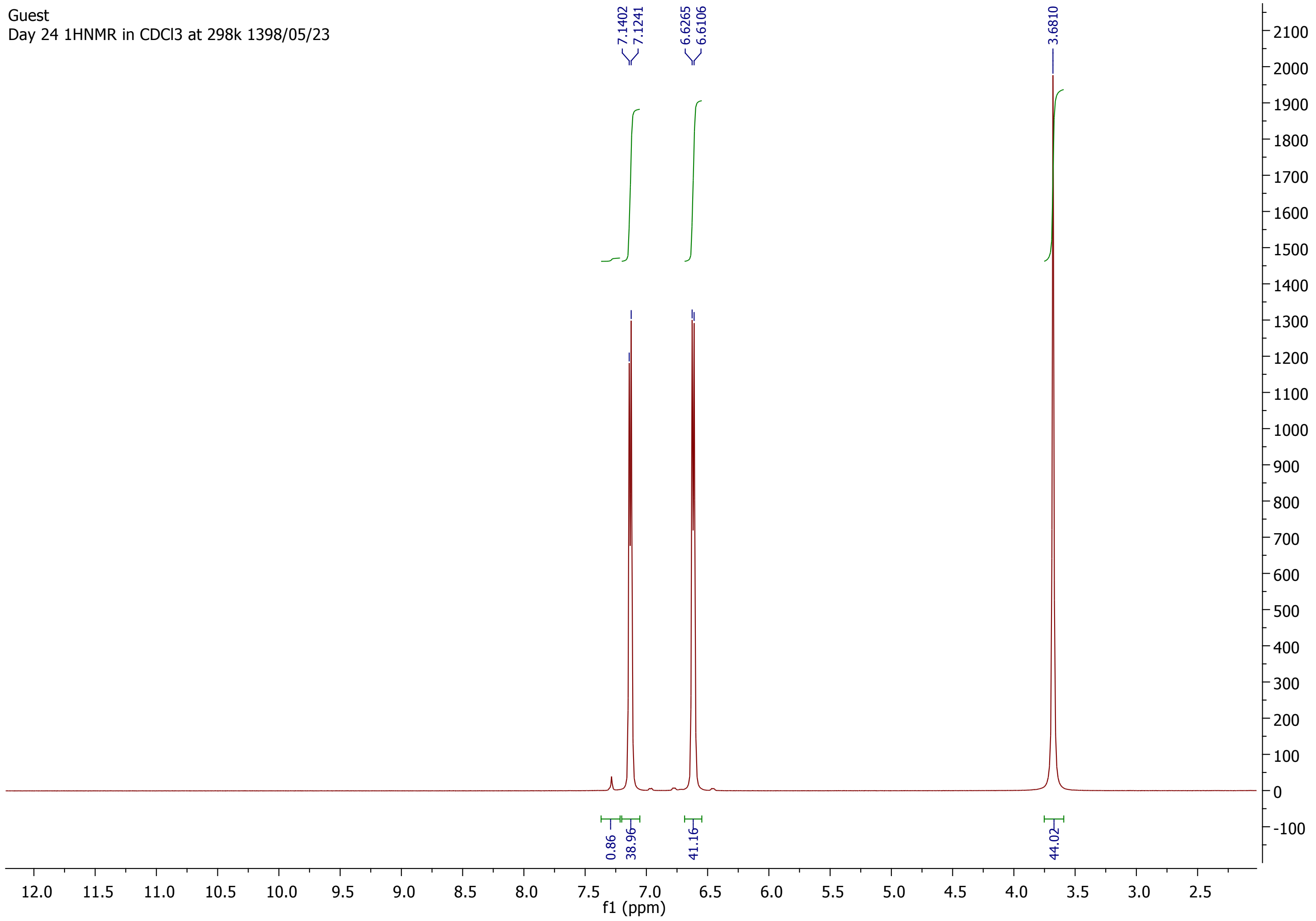

10. 4-chloroaniline **(Table 2– Entry 10)**

^{1}H NMR (500 MHz, CDCl3): δ 3.68 (s, 2H), 6.61-6.63 (d, J = 10 Hz, 2H), 7.12-7.14 (d, J = 10 Hz, 2H).

Figure S19. ^{1}H-NMR spectra and spectral data of 4-chloroaniline.

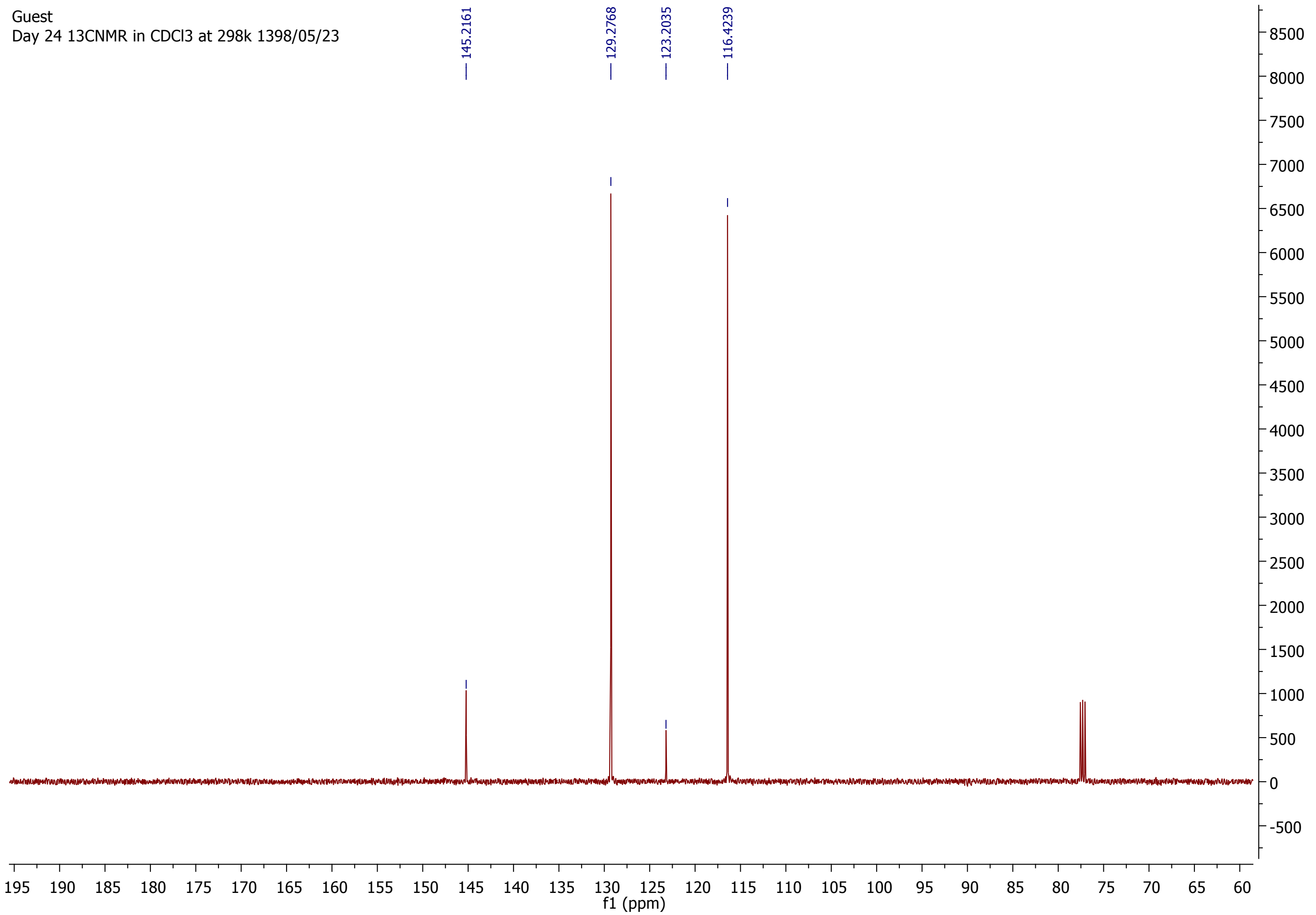

10. 4-chloroaniline (**Table 2– Entry 10)**

^{13}C NMR (500 MHz, $CDCl_3$): δ 116.42, 123.20, 129.28, 145.22.

Figure S20. ^{13}C-NMR spectra and spectral data of 4-chloroaniline.

Table S1. Results of ICP-OES analysis of Fe_3O_4@VPMP/CLS-Cu nanocomposite.

Element	Wavelength (nm)	LOD (mg/Kg)	RSD [a] (%)	ICP-OES (mg/g)
Na	588.995	1.01	9.11	2.0
Fe	259.940	0.0528	4.18	312.14
Al	167.081	0.459	12.6	26.52
Ca	422.673	0.263	5.43	3.06
K	769.896	3.12	15.6	2.24
Cu	324.754	0.0732	6.23	255.85

[a] *n=3.*

Estimation of the catalyst amount in mmol% based on ICP-OES results

Since, copper element is the main active catalytic site in the structure of the Fe_3O_4@VPMP/CLS-Cu nanocomposite, the weight ratio of Cu is considered for conversion to mmol% of the catalyst, as below;

Per 1000 mg of catalyst: 255.85 (mg Cu) = 0.255 (g Cu) = 4.01 (mmol Cu)

Based on Table 1 (in manuscript), 50.0 mg of Fe_3O_4@VPMP/CLS-Cu is subjected to the reaction mixture, under optimum conditions, so the mmol of Cu in 50.0 mg of Fe_3O_4@VPMP/CLS-Cu is calculated in 0.2 mmol (4.01/20).

Since, 1.0 mmol of the nitrobenzene (NB) derivatives is used for reduction;

0.2 (mmol Cu) / 1.0 (mmol NB) × 100 = **20 mmol%** (catalyst used in NB reduction reactions)

Calculation of turnover number (TON) and turnover frequency (TOF)

As described above, copper is the main catalytic site in the structure of Fe_3O_4@VPMP/CLS-Cu for reductive conversion of the nitrobenzene derivatives. Hence, the mmol% of the copper element in the optimum conditions (Table 1, in manuscript) can be considered for the estimation of TON and TOF values, as below:

Turnover number (TON) = number of moles of reactant consumed / mole of catalyst

Turnover frequency (TOF) = TON / time of reaction (h)

TON = 98 / 20 = 4.9

TOF = 4.9 / 0.133 = 36.84 h^{-1}

Estimation of half-life of the reduction reaction of nitrobenzene and investigation of reusability of Fe_3O_4@VPMP/CLS-Cu catalytic system:

The *half-life* of the reduction reaction of nitrobenzene (NB) as the time it takes for the concentration of a reactant to fall to half of its original value, can be estimated by UV-Vis spectroscopy.

For this purpose, a control reaction containing NB (20 mg/mL) in water under catalytic conditions provided by a dispersion of Fe_3O_4@VPMP/CLS-Cu particles (40 mg), $NaBH_4$ (62 mg), and K_2CO_3 (30 mg), was monitored in a range of time 0-8 minutes. For sampling at different times (2, 4, 6 and 8 min), the particles were magnetically collected by an external magnet and 5.0 µL of the mixture was withdrawn by a micropipette and diluted to 50 mL with water. Then, paper filtration was performed on the prepared samples and the prepared samples were studied by UV-Vis spectrophotometer in a wavelength range of 200-350 nm. Finally, the concentrations of the aniline and NB in the reaction mixture were calculated by the line equations obtained from the calibration curves (Figure S21a). As can be observed in Figure S21, considering the calibration curves of aniline and NB obtained from the standard solutions in different concentrations (panel a) and the provided UV-Vis spectra (panel b), the concentration balance between aniline and nitrobenzene UV-Vis spectroscopy has been created after **4 minutes** from the beginning of the reaction.

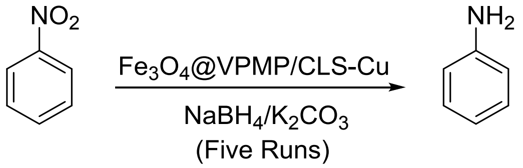

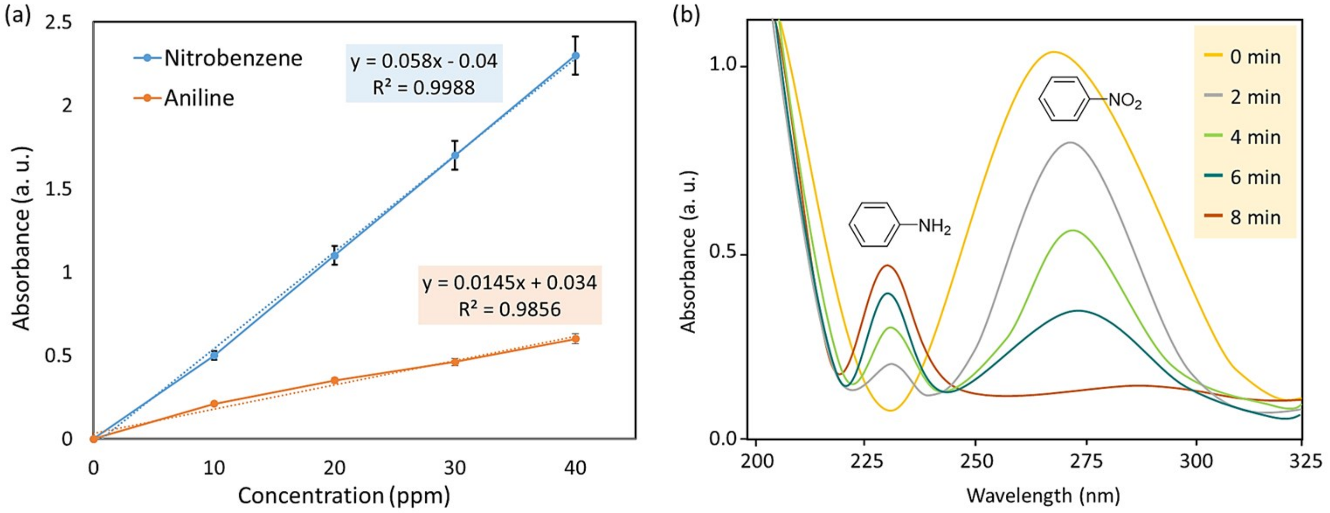

Figure S21. (a) Calibration curves of nitrobenzene (NB) and aniline solutions in different concentrations (error bars show %RSD in each point, n = 3), and (b) UV-Vis absorbance spectra of the NB reaction mixture, under catalytic conditions in different times.

Accordingly, the half-life values for the next successive runs in the recycling process have been estimated and reported in Table S2. As is seen in the table, the time of half-life of the reduction reaction of the NB has changed to higher values after each time of separation and re-preparation of the catalyst particles. Based on the obtained results, it can be concluded that the catalytic efficiency of the Fe_3O_4@VPMP/CLS-Cu system is gradually lost during the recycling and reusing processes. To investigate the possible reasons, probable leaching of the copper particles from the Fe_3O_4@VPMP/CLS-Cu system as the main catalytic site has been checked by inductively coupled plasma mass spectrometry (ICP-MS) analysis on the supernatant after the separation of the magnetic particles after running each recycling process. As is seen in Table S3, a bit of leaching has occurred for the copper particles during the separation, rinsing, drying and reusing of the Fe_3O_4@VPMP/CLS-Cu particles in successive five runs.

Table S2. The yields and half-life values obtained for the reduction reaction of nitrobenzene (NB), under catalytic conditions provided by Fe_3O_4@VPMP/CLS-Cu system during five times reuse.

Run	Half-life (min)	RSD [a] (%)	Reaction yield [b] (%)
1	4.0	5.0	98
2	4.3	4.8	94
3	4.5	6.6	91
4	4.6	3.7	89
5	4.8	7.1	86

[a] *Relative standard deviations have been estimated for three samples per each condition (n = 3);* [b] *Reaction time: 8 minute, isolated yields after purification of the aniline.*

Table S3. Results of ICP-MS analysis on copper ion exist in supernatants after separation of the magnetic particles, after running each recycling process (leaching test).

Run	LOD (ppm)	RSD [a] (%)	ICP-MS (ppm)
1	0.03	2.1	9.4
2	0.03	1.1	7.6
3	0.03	1.6	4.3
4	0.03	2.3	3.8
5	0.03	1.4	3.1

[a] *n=3.*

Table S4. Results of ICP-OES analysis of the recovered Fe_3O_4@VPMP/CLS-Cu nanocomposite.

Element	Wavelength (nm)	LOD (mg/Kg)	RSD [a] (%)	ICP-OES (mg/g)
Na	588.995	1.01	9.11	1.71
Fe	259.940	0.0528	4.18	299.29
Al	167.081	0.459	12.6	25.67
Ca	422.673	0.263	5.43	2.88
K	769.896	3.12	15.6	1.39
Cu	324.754	0.0732	6.23	207.12

[a] *n=3.*

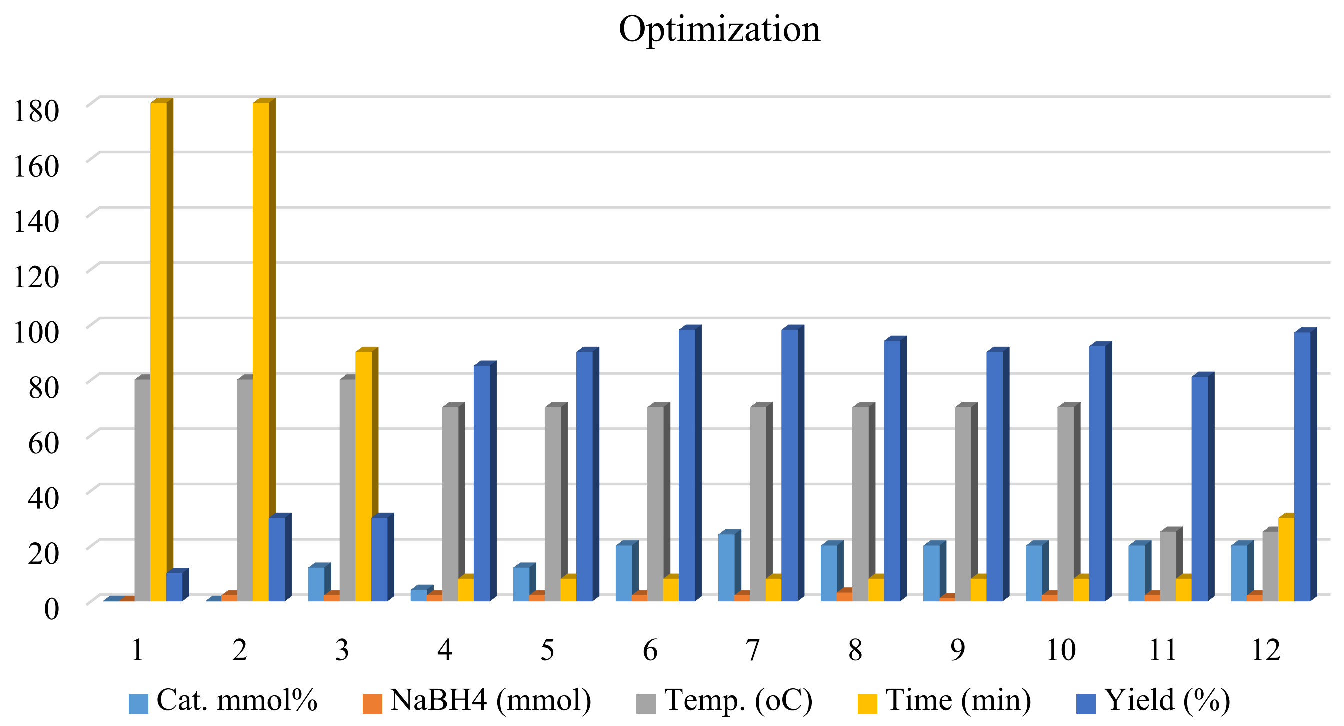

Figure S22. The graph of the optimization stages of reduction reaction of nitrobenzene under various catalytic conditions (related to Table 1).
